# Mining and Validation of Novel Umami Peptides in Non-Alcoholic Beer by Integrating Machine Learning Prediction, Molecular Docking, and Sensory Validation, and Their Multidimensional Sensory Impacts on Beer Body

**DOI:** 10.3390/foods15101671

**Published:** 2026-05-11

**Authors:** Yashuai Wu, Wenjing Tian, Zihan Shi, Yi Ren, Yiyuan Chen, Xin Yuan, Jiang Xie, Bofeng Zhong, Dongrui Zhao

**Affiliations:** 1 Department of Food and Bioengineering, Beijing Vocational College of Agriculture, Beijing 102442, China; wyss995418706@163.com; 2 School of Food Science and Engineering, South China University of Technology, Guangzhou 510640, China; 3 College of Business Administration, Kyung Hee University, Seoul 02447, Republic of Korea; shizihan62@khu.ac.kr; 4 Beijing Changping District Food and Drug Safety Surveillance Center, Beijing Changping District Market Supervision Administration, Beijing 102200, China; 409695694ryan@sina.com; 5 College of Food Science and Nutritional Engineering, China Agricultural University, Beijing 100083, China; chenyiyuan_1112@163.com; 6 Key Laboratory of Geriatric Nutrition and Health (Ministry of Education), Beijing Technology and Business University, Beijing 100048, China; yuanxin5215@163.com (X.Y.); xj2445580935@sina.com (J.X.); z125678hhh@outlook.com (B.Z.); zdr@btbu.edu.cn (D.Z.); 7 Key Laboratory of Brewing Molecular Engineering of China Light Industry, Beijing Technology and Business University, Beijing 100048, China; 8 National Baijiu and Huangjiu Research Institute, Beijing Technology and Business University, Beijing 100048, China; 9 China Food Flavor and Nutrition Health Innovation Center, Beijing Technology and Business University, Beijing 100048, China

**Keywords:** non-alcoholic beer, umami peptides, T1R1/T1R3, molecular docking, sensory validation

## Abstract

This study aimed to identify umami peptides in non-alcoholic beer and clarify their potential contribution to taste reconstruction and aftertaste improvement. Peptides were profiled by RPLC-Q-TOF-MS and screened using machine learning prediction, molecular docking, MM-GBSA analysis, and sensory validation. Under the criteria of −10logP ≥ 15 and ALC ≥ 90.00%, 2081 peptides were identified. Among them, 122 potential umami peptides were predicted, and 117 peptides were successfully docked with the T1R1/T1R3 umami receptor. The docked peptides were mainly short to medium oligopeptides, especially tetrapeptides and pentapeptides, which accounted for 40.17% and 35.90%, respectively. Based on docking score, structural diversity, and peptide length distribution, CTGAA, IDQILG, KDTHP, QRQ, and EITGR were selected as representative candidates. These peptides showed favorable receptor binding, mainly supported by hydrogen bonding, electrostatic interactions, and local hydrophobic contacts. Sensory validation further showed that the 5 peptides improved umami and aftertaste cleanliness to different degrees. Umami intensity increased by 7.58% to 22.73%, while aftertaste cleanliness increased by 5.80% to 17.39%. Among them, CTGAA showed the strongest umami enhancement, and QRQ produced the greatest improvement in aftertaste cleanliness. These results suggest that selected umami peptides may contribute to flavor reconstruction in non-alcoholic beer by enhancing umami perception and improving aftertaste quality.

## 1. Introduction

Beer is a complex fermented beverage containing ethanol, carbohydrates, minerals, vitamins, phenolic compounds, and hop-derived bitter substances, and its sensory appeal, potential health-related value, and safety are closely associated with both raw materials and brewing processes [1]. In the contemporary global consumer market, the development logic of non-alcoholic beer (NAB) is undergoing a profound paradigm shift. Early non-alcoholic beer was primarily positioned as an alcohol substitute for specific groups such as drivers or consumers with religious restrictions. Its production processes mainly focused on achieving compliant ethanol levels through physical approaches such as vacuum distillation, pervaporation, or restricted fermentation, usually defined as an alcohol content below 0.5% by volume [2,3,4]. However, although this subtractive technological pathway successfully reduced the physiological burden associated with ethanol intake, it also caused an overall loss of body structure at the sensory level. With the growing prevalence of health awareness and advances in precision brewing technology, non-alcoholic beer has moved beyond its single substitute role and has evolved into an independent technological pathway jointly driven by healthy consumption, functional orientation, and flavor design [5,6,7].

During the actual consumption of non-alcoholic beer, the most common contradiction lies not only in weakened aroma, but more importantly in a hollow body, insufficient support on the palate, loose sensory coherence, and a shallower aftertaste. For such products, flavor optimization that remains limited to volatile aroma is often insufficient to truly solve palatability problems [8,9,10]. Taste-active components, especially umami substances that can influence fullness, roundness, and persistence, have therefore become more worthy targets for in-depth study in efforts to improve the quality of non-alcoholic beer [2,11,12,13,14].

Compared with bitterness, sweetness, and aroma, umami has long remained at a relatively marginal position in beer research. This does not mean that umami lacks perceptual significance in beer. On the contrary, umami is often closely associated with body thickness, oral fullness, harmony, and post-consumption pleasantness. In complex fermentation systems, this role usually does not appear as a single high-intensity signal, but instead participates in the construction of overall taste perception in a background, supportive, and integrative manner [15,16,17]. This is particularly true for non-alcoholic beer. Because the contribution of alcohol is markedly weakened, the fullness and balance originally maintained in part by ethanol require compensation from other non-volatile components. From this perspective, the exploration of umami-active molecules is not simply an attempt to add one more taste, but an important entry point for reconstructing the sensory framework of non-alcoholic beer [18,19,20].

Current beer flavor research has accumulated substantial knowledge on esters, higher alcohols, organic acids, isomerized bitter substances, sugars, and polyphenols, and has gradually begun to address the contribution of non-volatile taste factors such as amino acids and nucleotides to mouthfeel structure. In contrast, research at the peptide level remains insufficient [13,21,22,23]. Peptides in beer are not marginal components. They are involved throughout multiple stages, including malt protein degradation, saccharification, boiling reactions, and yeast fermentation. They influence foam, turbidity, and colloidal stability, and may also directly participate in taste expression. The problem is that beer peptides are highly diverse, broadly distributed, markedly different in abundance, and readily masked by background sweetness, saltiness, acidity, and bitterness [24,25,26,27,28]. In traditional studies, much of the work has focused more on their nutritional value, bioactivity, or processing properties, whereas truly taste-functional peptides have lacked systematic screening. Although recent studies on beer and other fermented foods have begun to identify umami peptides and apply machine learning tools for peptide prediction, most of these works have mainly focused on candidate discovery, taste prediction, or receptor-level interpretation rather than verifying their actual contribution within a defined non-alcoholic beer matrix. Therefore, the present study is distinguished from previous work by integrating peptide identification, machine-learning-assisted screening, sensory validation, and matrix-based contribution analysis to determine whether predicted peptides truly act as umami contributors in NAB. In particular, in the non-alcoholic beer system, the identification and validation of novel umami peptides still lag significantly behind [29,30].

Based on the above, and given the large number of candidate peptides in complex fermentation matrices, RPLC-Q-TOF-MS was used in this study to achieve high-resolution peptide detection and sequence-level identification in complex samples, thereby providing strong structural information support for the establishment of a candidate peptide library. In addition, machine learning prediction was employed to construct discriminative models based on the sequence characteristics of known umami peptides, enabling the rapid identification of more promising target molecules from a large-scale candidate peptide library and significantly reducing the resource consumption caused by blind synthesis and one-by-one validation. Molecular docking was further applied to provide structural interpretation at the level of receptor recognition. Umami perception is closely related to interactions with taste receptors. The binding conformation of candidate peptides with the umami receptor, as well as their hydrogen-bonding network and compatibility with key sites, can provide more targeted evidence for their potential umami activity. It should be emphasized that computational results still cannot replace real sensory conclusions. Beer is not a simple buffer system. Its acidity, bitterness, residual sugars, ionic strength, carbon dioxide state, and matrix interactions can all alter the actual taste performance of the same peptide. Therefore, representative novel umami peptides were finally selected, and each target peptide was individually added under a unified sample background to more clearly identify the independent contribution of a given umami peptide to umami intensity, sensory harmony, and potential off-flavor in non-alcoholic beer.

This study addresses this gap by moving beyond the simple prediction or annotation of beer peptides. It reports new umami peptide candidates in NAB and builds a targeted computational pipeline that combines peptide profiling, umami prediction, molecular docking, and binding energy evaluation. More importantly, these predicted peptides are not treated as theoretical results alone. They are further examined through sensory validation in a defined NAB matrix. This design links sequence information, receptor interaction, and real taste perception in the same study. It also offers a clearer route for identifying taste-active peptides that may contribute to the flavor construction of NAB.

## 2. Materials and Methods

### 2.1. Samples and Reagents

The experimental sample was a commercial Japanese non-alcoholic beer-taste beverage, Asahi Dry Zero, produced by Asahi Breweries, Japan. According to the manufacturer, this product is designed as an alcohol-free beer-like beverage with 0.00% alcohol, 0 sugar, and 0 calories. Unlike conventional NAB obtained by limited fermentation or post-fermentation dealcoholization, Asahi Dry Zero is produced using a complete blending process without wort fermentation. Beer-like flavor is reconstructed through formula design and flavor adjustment, which provides a relatively stable non-fermented matrix for evaluating peptide-related sensory changes. The labeled ingredients included dietary fiber, soy peptide, hops, carbon dioxide, flavoring, acidulant, caramel color, antioxidant, and sweetener. Therefore, the sample used in this study was not treated as a traditional fermented beer matrix, but as a representative commercial alcohol-free beer-taste beverage with a formulated beer-like flavor profile. After collection, all samples were purchased from the same commercial batch whenever possible. The product name, manufacturer, package type, production lot number, expiration date, and purchase date were recorded to reduce uncertainty caused by batch variability. Samples with damaged packaging or abnormal appearance were excluded. The samples were immediately refrigerated at 0 °C, and the sampling time was recorded using 24 h as 1 d. Before analysis, the samples were gently mixed and equilibrated under the same temperature conditions. This procedure was used to minimize variation caused by storage, carbonation loss, and package-to-package differences.

The core solvents used in the experiment were acetonitrile (ACN, ≥99%, chromatographic grade) and formic acid (FA, ≥99%, chromatographic grade), both purchased from Beijing Innochem Technology Co., Ltd. (Beijing, China). Ultrapure water was used throughout. The umami peptides used were CTGAA, IDQILG, KDTHP, QRQ, and EITGR (purity ≥ 90%, Shijiazhuang Langandun Trading Co., Ltd., Shijiazhuang, China).

### 2.2. Instruments

The major instruments and consumables used in this study included the following categories. Ultrapure water was prepared using a Milli-Q ultrapure water system (MilliporeSigma, Burlington, MA, USA). Routine pipetting and volume adjustment were performed using a 1000 μL pipette (Sinopharm Chemical Reagent Beijing Co., Ltd., Beijing, China) and 10- and 100 mL volumetric flasks (Weiye Metrology and Technology Research Group Co., Ltd., Zhengzhou, China). The consumables used for sample transfer and instrumental analysis were 2 mL autosampler vials (Beijing Yinuokai Technology Co., Ltd., Beijing, China). During sample mixing and pretreatment, a VM-500S vortex mixer (Qunan Scientific Instrument (Zhejiang) Co., Ltd., Zhejiang, China) and an SHB-III circulating water multifunction vacuum pump (Zhengzhou Greatwall Scientific Industrial and Trade Co., Ltd., Zhengzhou, China) were used. Sample clarification and low-temperature centrifugation were performed using a Fresco 17 refrigerated microcentrifuge (Thermo Fisher Scientific, Waltham, MA, USA). The analytical platform consisted of an ultra-performance liquid chromatography system (Waters Corporation, Milford, MA, USA) and a TripleTOF 5600 series QTOF high-resolution mass spectrometer (SCIEX, Marlborough, MA, USA).

### 2.3. Experimental Methods

#### 2.3.1. Pretreatment Method

The sample described in this subsection refers to the peptide-enriched reconstituted extract obtained after preliminary treatment of the original NAB, rather than the untreated beverage taken directly from the bottle. Before peptide extraction, the original NAB was degassed at low temperature and filtered to remove foam, suspended particles, and visible insoluble materials. The filtrate was then subjected to preliminary enrichment and cleanup, such as solid-phase extraction, to obtain a peptide-containing extract. The collected extract was concentrated by freeze-drying and then reconstituted with a small volume of ultrapure water or aqueous solvent. This reconstituted extract was used for the subsequent centrifugation step.

A 400 μL aliquot of the sample was transferred into a 1.5 mL centrifuge tube and vortexed for 30 s to ensure complete mixing. Before aliquoting, the beer sample was gently mixed and equilibrated to 20 °C. Obvious foam and sediment were avoided during sampling to reduce variation caused by carbon dioxide release and suspended particles. No additional enzymatic hydrolysis was introduced during pretreatment, so the detected peptides mainly represented endogenous peptides already present in the beer matrix. The sample was then centrifuged at 20 °C and 17,000× *g* for 15 min. The clarified supernatant was carefully collected and transferred to a new centrifuge tube without disturbing the bottom precipitate. This step was used to remove insoluble proteins, colloidal particles, yeast residues, and other suspended materials that could interfere with chromatographic separation and MS ionization. The obtained supernatant was dried in a vacuum centrifugal concentrator at 35 °C. The relatively mild drying temperature was selected to limit additional thermal degradation or oxidation of small peptides. The residue was then reconstituted with 100 μL of 75% (*v*/*v*) aqueous methanol, vortexed for 60 s, and ultrasonicated for 10 min to improve sample dissolution efficiency. The water-bath temperature was controlled during ultrasonication to reduce additional sample variation caused by temperature increase. The reconstituted solution was centrifuged again at 20 °C and 17,000× *g* for 10 min, and the supernatant was transferred into an autosampler vial for subsequent instrumental analysis. The final supernatant was used directly for RPLC-Q-TOF-MS analysis. No reduction, alkylation, or tryptic digestion was performed, because the aim of this study was to profile naturally occurring beer peptides rather than artificially generated enzymatic peptides.

#### 2.3.2. RPLC-Q-TOF-MS Analytical Conditions

Peptide separation was performed under reversed-phase liquid chromatography conditions using an ACQUITY UPLC HSS T3 column (C18, 1.8 μm, 2.1 mm × 100 mm, 100 Å, Waters, USA). The mobile phase consisted of an acidified aqueous phase and an acidified organic phase. Solvent A was 0.1% (*v*/*v*) aqueous FA, and solvent B was 0.1% (*v*/*v*) FA in ACN. The use of FA improved chromatographic peak shape and enhanced the positive ion response of peptide ions. The injection volume was 4 μL, the flow rate was 0.20 mL/min, and the total run time was 60 min. The gradient elution program was as follows. From 0.0 to 3.0 min, solvent B was maintained at 5%. From 3.0 to 5.0 min, solvent B increased linearly from 5% to 10%. From 5.0 to 34.0 min, solvent B increased from 10% to 20%. From 34.0 to 50.0 min, solvent B increased from 20% to 40%. From 50.0 to 56.0 min, solvent B increased from 40% to 95% and was briefly held. After 56.1 min, the system was returned to 5% B and equilibrated until the end of the run. This long and shallow gradient was used to improve the separation of beer peptides with similar hydrophobicity and to reduce co-elution in the complex beer matrix.

Mass spectrometric detection was performed using a TripleTOF 5600 QTOF system controlled by Analyst TF 1.7 software. Data were acquired in information-dependent acquisition (IDA) mode, which is essentially a data-dependent acquisition strategy. In each acquisition cycle, an MS^1^ full scan was first performed as the survey scan. Up to 10 precursor ions with signal intensities above 100 cps were then selected for MS^2^ fragmentation analysis. The MS^1^ scan range was set at *m*/*z* 100–1500, and the collision energy was 30 eV. The IDA mode was selected to obtain both accurate precursor ion information and product ion spectra for subsequent peptide sequencing and database matching. The electrospray ion source was operated in positive ion mode with the following settings: GS1 60 psi, GS2 60 psi, CUR 35 psi, ion source temperature 550 °C, and ion spray voltage 5500 V.

#### 2.3.3. Qualitative Identification and Analysis of Peptides in Beer

Peptide identification and precursor protein source assignment were performed using the PEAKS Studio platform. Because the beer peptidome originated from both cereal raw materials and fermentative microorganisms, an integrated reference proteome strategy was adopted to approximate the actual protein composition of the sample as closely as possible. FASTA sequences from the UniProt reference proteomes of *Hordeum vulgare*, *Triticum aestivum*, and *Saccharomyces cerevisiae* were merged to construct a custom search database. These 3 organisms were selected because barley and wheat were the main cereal-related protein sources, while Saccharomyces cerevisiae represented the major fermentative microorganism contributing yeast-derived peptides and proteins. UniProt still organizes reference proteomes as standardized and downloadable data units and updates them regularly. It is therefore suitable as a basic sequence set for source assignment in complex food systems.

Considering that endogenous beer peptides are mainly generated through the coordinated action of malt, yeast, and multiple proteases that persist throughout brewing, their cleavage sites usually do not follow classical specific enzymatic digestion rules such as those of trypsin. Therefore, the enzyme mode was set to no enzyme in the database search, and unspecific digestion was used for matching. Missed cleavage was not used as a restrictive parameter under this search mode. At the same time, the de novo sequencing module was enabled, and the resulting sequence tags were used for subsequent database matching to enhance the identification of peptides that were insufficiently covered by the database, peptides from low-coverage regions, and potential variant sequences.

The mass tolerance parameters were set to 10 ppm for precursor ions and 0.03 Da for fragment ions to match the mass accuracy level of the high-resolution QTOF-MS data. The precursor charge state was assigned according to the acquired MS data and the PEAKS search workflow. Only peptide candidates supported by matched MS^2^ fragment ions and falling within the defined mass error window were retained for further screening.

For modification settings, common types of chemical modifications in food sample processing and natural peptides were comprehensively considered, while the effect of search-space expansion on identification specificity was also taken into account. Oxidation of methionine, peptide N-terminal acetylation, N-terminal pyroglutamylation of glutamine or glutamic acid, and deamidation of asparagine/glutamine were set as variable modifications. These modifications are common structural changes in peptidomics analysis. Among them, methionine oxidation and deamidation may reflect the natural state of the sample or may arise during sample preparation and analysis. They were therefore included in the search as variable modifications. If reduction and alkylation were used during pretreatment, the corresponding cysteine modification was further included in the search parameters. Because no reduction and alkylation were performed during sample pretreatment, carbamidomethylation of cysteine was not set as a fixed modification in the main search. This setting avoided introducing an artificial modification that was not generated by the experimental workflow.

The identification results were statistically validated using a target-decoy strategy, and the false discovery rate was controlled at 1% at both the peptide-spectrum match and protein levels. Peptide-spectrum matches that did not pass the FDR threshold were excluded. Peptides with poor MS^2^ spectral support, unreasonable mass deviation, or ambiguous sequence assignment were not used for subsequent umami peptide prediction. The final peptide list therefore represented a filtered set of endogenous beer peptides supported by high-resolution MS and database-assisted de novo identification. Methodological studies related to PEAKS have shown that the results generated by de novo-assisted searching still require stringent FDR filtering under a target-decoy framework to maintain reliability while improving identification coverage [31,32,33].

#### 2.3.4. Efficient Screening of Potential Umami Peptides Using Machine Learning

To efficiently screen potential umami peptides from large-scale candidate peptide sequences and provide prioritized candidates for subsequent molecular docking, receptor interaction analysis, and sensory validation, a machine-learning prescreening step based on primary structure information was introduced before experimental validation. It should be noted that the present study did not retrain these models. Instead, 2 previously established online predictors were used as external screening tools, and their published training sources, validation strategies, and evaluation indicators were described to clarify the reliability and boundary of the prediction results. First, the candidate peptide sequences were standardized according to the input requirements of the models, and sequence format conversion was completed using a peptide FASTA format converter, as described in Supplementary S1. At the same time, a peptide chemical symbol calculator was used to assist in organizing and checking the chemical composition information of the candidate sequences, as described in Supplementary S2.

On this basis, the standardized candidate peptide sequences were separately entered into the online prediction platform of the Umami-Transformer model [34] and the umami peptide prediction tool built on the iUmami-SCM model [35] (Supplementary S3) for parallel prediction analysis. For Umami-Transformer, the original model was developed from a curated umami peptide dataset and combined a Transformer-based sequence representation with 8 physicochemical descriptors. Its reported performance was evaluated using classification metrics including accuracy, F1 score, and Matthew’s correlation coefficient, with reported values of 0.965, 0.903, and 0.889, respectively. The Umami-Transformer model can learn contextual association information among residues from amino acid sequences, thereby enabling high-accuracy identification of umami peptides.

For iUmami-SCM, the original training and testing datasets were derived from known umami and non-umami peptide sequences. The training dataset UMP-TR contained 112 umami peptides and 241 non-umami peptides, while the independent dataset UMP-IND contained 28 umami peptides and 61 non-umami peptides. The model was developed using a 10-fold cross-validation strategy and further examined with an independent test set. Its performance was assessed using common binary classification indicators, including accuracy and Matthew’s correlation coefficient, and the independent test accuracy and Matthew’s correlation coefficient were reported as 0.865 and 0.679, respectively. The iUmami-SCM model discriminates candidate peptides based on sequence composition features and dipeptide propensity scores.

For each candidate peptide sequence, the predicted class and corresponding score returned by the 2 models were recorded separately, and the results were summarized. By introducing a dual-model parallel prescreening strategy, the influence of prediction bias from a single model on the screening results could be reduced to some extent, and cross-evidence could be provided for the subsequent selection of candidate umami peptides. Only peptides with positive prediction results or relatively high prediction scores in at least 1 model were retained as priority candidates, while peptides supported by both models were given higher screening priority. This rule was used to avoid treating computational prediction as direct sensory evidence. The final judgment still depended on subsequent molecular docking, receptor interaction analysis, and sensory validation in the non-alcoholic beer matrix. Finally, the candidate peptides were prioritized according to the prediction results and scores generated by each model, thereby improving the targeting and screening efficiency of subsequent validation work.

#### 2.3.5. Molecular Docking Method

Considering that T1R1/T1R3 is regarded as the principal umami receptor, a three-dimensional model of its extracellular ligand-binding domain was constructed using a homology-modeling strategy and was used for subsequent molecular docking analysis. First, the reference amino acid sequences were obtained from UniProtKB as the target sequences. The structure 1EWK was then selected from the RCSB PDB as the template. This structure corresponds to the experimentally resolved extracellular ligand-binding region of mGluR1 in complex with glutamate and can provide reliable reference information for binding pocket localization, local conformational constraints, and the spatial arrangement of key residues [36,37]. It was therefore used as the starting template for subsequent homology modeling. Modeling was completed on the SWISS-MODEL online platform using the target sequences and the 1EWK template as inputs. Following the general homology-modeling procedure, template identification, target-template structural alignment, three-dimensional model construction, and model quality evaluation were performed sequentially [38,39]. During modeling, special attention was paid to the conservation between the target sequence and the template sequence in the ligand-binding region and adjacent secondary-structure regions. The distribution of insertion and deletion fragments at loop regions and pocket edges was also examined to reduce local folding bias and pocket geometry artifacts caused by misalignment. For regions directly related to orthosteric ligand binding, priority was given to maintaining reasonable backbone conformations and side-chain orientations, thereby improving the structural reliability of subsequent docking results [39,40]. After model generation, structural quality control was performed using the SAVES v6 platform, and the distribution of backbone dihedral angles was further evaluated using the PROCHECK/Ramachandran plot. Particular attention was paid to whether residues were mainly distributed in the favored and allowed regions and whether obvious abnormal conformational residues were present [41].

After the validated T1R1/T1R3 model was loaded into Maestro 14.5, standard preprocessing was completed using the Protein Preparation Wizard. This workflow included the addition of hydrogen atoms, correction of bond orders and atomic valences, normalization of the chemical states of residues and hetero groups, optimization of the hydrogen-bond network, and restrained minimization under the OPLS4 force field to relieve local bond-angle strain and steric clashes that may have been introduced during modeling or hydrogen addition. Non-critical hetero components in the receptor structure were in principle removed. However, if specific crystal water molecules, ions, or other auxiliary components were judged to participate in pocket stabilization or ligand recognition, they were retained to avoid excessive simplification of the local binding environment [36,40].

The three-dimensional structures of the candidate peptide ligands were generated jointly by LigPrep and Epik. LigPrep was used to convert the input structures into high-quality three-dimensional conformations suitable for structural biology calculations and to systematically enumerate possible tautomers, ionization states, ring conformations, and necessary stereochemical forms. Epik was used to predict pKa values and protonation-state distributions under aqueous conditions. In this study, the solution condition was set to pH 7.0 ± 2.0 to cover as fully as possible the major protonation and tautomeric states of the candidate peptides in near-neutral systems, thereby reducing the interference caused by unreasonable charge assignment and improving the consistency of ligand input states. The receptor grid was generated with the glutamate-binding region indicated by the template structure 1EWK as the center and was appropriately extended to cover the edges of the binding pocket and adjacent accessible regions, so that the candidate peptides had sufficient sampling space near the orthosteric site. Glide was then used for ligand sampling and scoring. The SP mode was used at the initial screening stage to balance sampling efficiency and conformational discrimination. Conformations with top scores or representative binding modes were further re-evaluated using the more stringent XP mode. After docking, the low-energy binding pose of each candidate peptide was retained, and clustering based on heavy-atom root-mean-square deviation was performed to identify representative binding conformations for subsequent visualization and interaction-pattern analysis. The analysis mainly included hydrogen bonds, salt bridges, electrostatic interactions, hydrophobic/van der Waals contacts, and possible cation–π interactions [41,42,43].

#### 2.3.6. MM/GBSA Binding Free Energy Calculation

To further improve the discrimination of docking results and compare the binding stability of complexes formed between different candidate peptides and the T1R1/T1R3 receptor from an energetic perspective, the MM-GBSA workflow in the Prime module was called within the Maestro v14.5 environment to estimate the endpoint binding free energies of the docked T1R1/T1R3-peptide complexes. MM-GBSA is essentially a post-processing rescoring strategy that couples molecular mechanics energy terms with the generalized Born implicit solvent model and a nonpolar surface area term. It is commonly used for rapid comparison of ligand-binding trends and prioritization under conditions of a unified receptor conformation and the same binding site. Because it balances computational efficiency and conformational discrimination, it remains widely used as a post-docking screening tool in recent receptor-ligand screening and peptide-receptor interaction studies [44,45].

Specifically, MM-GBSA calculations were initiated from the complex conformations obtained by molecular docking. First, local and restrained geometry optimization was performed on the complex to relieve local bond length, bond angle, or atomic contact conflicts that may have been introduced during docking, while preserving as much as possible the overall receptor backbone and the acquired binding mode. Subsequently, the free-energy terms of the optimized complex, the free receptor, and the free ligand were calculated under a unified implicit solvent environment, and the estimated binding free energy was obtained according to

ΔG_bind_ = G_complex_ − G_receptor_ − G_ligand_.

#### 2.3.7. Sensory Evaluation

All experimental samples were obtained from the same product batch, and the original beer sample was labeled A. On this basis, the five representative umami peptides, CTGAA, IDQILG, KDTHP, QRQ, and EITGR, were individually added to the original beer sample. Specifically, 4 mg of each standard umami peptide sample was added to 200 mL of the original beer sample to ensure that only the representative umami peptide affected the sensory properties of the beer body. The peptide-added beer samples were labeled A-1, A-2, A-3, A-4, and A-5, respectively. All samples were prepared immediately before sensory evaluation. They were gently mixed to ensure complete peptide dissolution and then equilibrated at 4 °C before serving. Each sample was poured into a clean transparent sensory cup and coded with a random 3-digit number. The serving order was randomized for each assessor to reduce order effect and psychological expectation.

The sensory panel consisted of 10 assessors, all of whom had received standardized sensory evaluation training. Panelists were selected from individuals with previous experience in beer sensory evaluation. They had normal taste and olfactory sensitivity, no reported allergy to beer ingredients, and no smoking, eating, or drinking of strongly flavored foods within 1 h before the test. Before formal evaluation, the panelists were trained using representative beer samples and reference solutions for sweetness, sourness, bitterness, umami, body fullness, carbonation sensation, and aftertaste. The training focused on attribute recognition, scale use, score repeatability, and consensus building. Only assessors who could consistently identify the target sensory attributes and showed stable scoring behavior in preliminary sessions participated in the formal test.

For the sensory evaluation of NAB, in addition to umami intensity, systematic assessment was carried out across four primary dimensions, namely appearance, aroma, mouthfeel, and drinking experience (Supplementary S7). Each sensory attribute was evaluated using a 0 to 10 intensity scale, where 0 indicated not perceptible and 10 indicated extremely strong. For overall liking and drinking acceptance, a hedonic score was also recorded, with higher values indicating stronger preference. The intensity scale was used to describe the sensory changes caused by peptide addition, while the hedonic score was used to judge whether these changes improved the actual drinking experience. This is because the quality changes caused by limited fermentation, dealcoholization, or membrane separation in NAB are not limited to weakened umami, but are often also manifested as reduced aroma complexity, insufficient body support, thin mouthfeel, and lower overall drinking pleasure [18,20,46,47].

Previous studies have shown that ethanol is not only an important component of the beer flavor system but also affects the partitioning and release of volatile compounds in the matrix and is closely related to body fullness, warming sensation, and aftertaste persistence. Therefore, when ethanol levels are reduced, NAB often shows varying degrees of decline in aroma expression, mouthfeel thickness, and the integrity of the drinking experience. On this basis, although the individual addition of umami peptides mainly aims to improve umami expression, their sensory effects are usually not limited to umami itself. Recent studies have suggested that when umami-active substances enter complex food systems, they are often accompanied by changes in thickness, roundness, persistence, and overall flavor harmony, and may exert coordinated modulation on sweetness, saltiness, bitterness, and the overall flavor tone. Therefore, evaluation of umami alone makes it difficult to comprehensively judge the true role of umami peptides in NAB or determine whether they further improve or weaken mouthfeel balance and the overall drinking experience. On this basis, simultaneous evaluation of mouthfeel and drinking experience helps to more accurately reveal the contribution of umami peptides to the overall flavor reconstruction of NAB.

During formal evaluation, each assessor independently evaluated all coded samples under controlled sensory conditions. The samples were served at the same temperature and evaluated under consistent lighting. Drinking water was provided for palate cleansing between samples. A rest interval was arranged between samples to reduce the carry-over effect, especially for bitterness, umami, and aftertaste attributes. Each sample was evaluated in duplicate, and the mean score of the repeated evaluations was used for subsequent analysis.

During sensory evaluation, for uncertain scores in specific sensory dimensions, an objective scoring tool for beer sensory evaluation (Supplementary S4) was used. The specific descriptions provided by the assessors for the corresponding beer sample were entered into this tool to obtain the score for the corresponding sensory dimension. The beer sensory evaluation objectification tool developed in this study was based methodologically on descriptive sensory analysis and quantitative descriptive analysis. Using 5000 online beer reviews as the basic corpus (Supplementary S5), professional beer sensory assessors combined actual post-consumption perceptions with consumer review texts to assign standardized scores to multidimensional beer attributes, including appearance, aroma, taste, and post-drinking sensation, thereby constructing a training dataset linking consumer descriptions with expert scores. Such methods are conducted by trained assessors who discriminate, describe, and quantify clearly defined sensory attributes using a unified intensity scale.

The final sensory data were expressed as mean ± standard deviation. One-way analysis of variance was used to compare sensory differences among the original beer and peptide-added samples. When significant differences were observed, multiple comparison analysis was performed to determine which samples differed from each other. The significance level was set at *p* < 0.05. Principal component analysis was further conducted using the mean sensory scores of each attribute to visualize the overall sensory distribution of the samples and to identify the main attributes contributing to sample separation.

Variability among panelists was also considered. For each sensory attribute, individual scores were examined before statistical analysis. The coefficient of variation was calculated to describe the dispersion of scores among assessors. If an assessor showed abnormal scoring patterns, poor repeatability between duplicate evaluations, or clear deviation from the panel consensus across most attributes, the data were checked together with the original sensory sheet. This step was not used to artificially remove disagreement, because individual perception is part of sensory evaluation. Rather, it was used to distinguish normal perceptual variability from operational error or misunderstanding of the attribute definition. Through this procedure, the reliability of the trained panel data and the interpretability of peptide-induced sensory changes were improved.

#### 2.3.8. Statistical Analysis

Statistical analyses were mainly performed in the R 4.5.x environment. For multiple-comparison problems, appropriate corrections were applied according to the specific comparison framework, and adjusted *p* values were consistently reported. To improve the reproducibility of statistical inference, key hypothesis testing and regression analyses were further cross-validated in the Python v3.10.9 environment using independent scripts. For data conforming to a one-factor design, one-way analysis of variance was performed using IBM SPSS Statistics 24, and *p* < 0.05 was used as the criterion for statistical significance. In the correlation analysis, *r* > 0.8 was used as the threshold for a strong positive correlation. Data visualization and curve fitting were performed in GraphPad Prism v10.0.0 and OriginPro 2021, respectively. The former was mainly used for routine statistical graphing and result presentation, whereas the latter was mainly used for linear or nonlinear curve fitting and goodness-of-fit testing. Multidimensional visualizations, including heatmaps and clustering plots, were generated using TBtools-II v2.303.

## 3. Results and Analysis

### 3.1. Qualitative Identification of Peptides in NAB and Analysis of Their Potential Contribution to Umami Flavor

In this study, a confidence threshold of −10lgP ≥ 15 was applied to the peptides obtained from database searching. For the de novo results, only sequences with ALC ≥ 90% were retained to ensure sequence reliability. A total of 2081 peptides were identified (Supplementary S6).

For the present dataset, each peptide was assigned 2 independent prediction outputs, including the predicted class and the corresponding confidence score. A peptide was retained as a potential umami peptide only when it was classified as umami and showed a prediction score higher than 0.7 in at least 1 model. This 0.7 cutoff was selected as a conservative high-confidence threshold rather than a default binary boundary. It was used to reduce false-positive retention from weak predictions. Peptides supported by both models were considered higher-priority candidates. Peptides supported by only 1 model were not excluded immediately, but were treated as lower-confidence candidates and were required to pass subsequent docking and sensory evaluation steps. To examine whether candidate selection was overly dependent on this cutoff, the screening results were also checked under nearby score boundaries. The main high-ranking candidates remained stable, indicating that the final selection was not driven by an arbitrary threshold alone.

Under this rule, 122 potential umami peptides were identified (Table 1). These peptides should be understood as computationally prioritized candidates rather than confirmed umami-active peptides. Their final relevance was further examined through receptor docking, interaction analysis, and sensory validation in the non-alcoholic beer matrix. This multi-step strategy reduced the uncertainty caused by single-model prediction and provided a more transparent basis for candidate peptide selection.

As shown in Supplementary S6, this peptide library exhibited the typical characteristics of low molecular weight, strong heterogeneity, and multiple origins. A total of 2081 unique sequences were obtained. Peptide lengths ranged from 2 to 40 amino acid residues, with an average length of 5.73 aa. Short peptides of 3 to 5 aa accounted for 68.19%, and sequences of 2 to 8 aa accounted for 88.19%, indicating that the peptides in this sample were mainly oligopeptides generated after extensive hydrolysis. In terms of amino acid composition, P, Q, G, I, V, and A showed the highest occurrence frequencies, accounting for 11.78%, 10.25%, 8.76%, 8.47%, 7.70%, and 6.61% of total residues, respectively. This result indicated that the peptide library contained both repetitive fragments rich in proline and glutamine and hydrophobic backbones represented by isoleucine, valine, and alanine. At the sequence level, a large number of short motifs such as QQ, QP, IP, GP, and LP were observed. Clear repetitive or extended fragments, including GQGQQG, QQAELIIPQQPQQP, QLEATTSIALRTLP, and QLNPCKVFLQQQCSPV, were also detected. These features were highly consistent with the Q-rich and P-rich repetitive structures commonly found in barley hordein and wheat prolamin storage proteins, and they also suggested that some peptides retained the structural signatures of the original storage proteins during brewing. Meanwhile, hydrophobic residue-rich sequences such as AAGFAG, AVGIGNVF, VVGFAFYSGF, FVRILVTP, and LVDFVANHPFLFLIREDIAGVVVFVGHVTNPLISA were present at relatively high proportions, indicating that this peptide library was not composed solely of Q/P repetitive fragments but also contained relatively hydrophobic segments derived from brewing raw-material proteins and yeast proteins. A considerable number of sequences also contained C, M, H, K, or R, such as EQHQLNLCKEFLLQQCT, GDRQTVCNCLK, GECCNGVRDLHNQ, and TKGRR, reflecting the high complexity of this peptide library in terms of sulfur-containing residues, basic sites, and potentially oxidation-sensitive sites. Overall, these peptides did not constitute a homogeneous short-chain collection. Instead, they consisted of a large number of short oligopeptides, some repetitive Q/P-rich fragments, and a small number of longer fragments that were relatively resistant to further degradation. They retained the sequence features of cereal storage proteins and also reflected the structural diversity jointly shaped by continuous proteolysis and multiple protein origins during brewing. Beer peptidomics studies have shown that peptides in beer mainly originate from barley and, to a lesser extent, from wheat and yeast, whereas continuous proteolysis during brewing releases a large number of short peptides [48,49,50]. In addition, hordein, as a representative barley prolamin, is itself characterized by high P, high Q, and repetitive motifs, which is consistent with the overall distribution pattern of the sequence set obtained in this study [51,52].

Based on the potential umami peptide sequences listed in Table 2, the 122 potential umami peptides screened in this study exhibited prominent short-chain and structurally concentrated features. Peptide lengths ranged from 2 to 8 aa, with an average length of 4.52 aa. Among them, 47 and 42 peptides were 4 aa and 5 aa in length, accounting for 38.52% and 34.43%, respectively, for a combined proportion of 72.95%. This indicated that these candidate sequences were mainly composed of short oligopeptides. Further examination of the primary structures showed that the residue composition was not evenly distributed. A, P, I, S, E, T, D, and V were the dominant amino acids, among which A, P, and I accounted for relatively high proportions, indicating that the sequence backbone combined small molecular size, a certain degree of hydrophobicity, and local conformational constraint. From the perspective of functional sites, 65 sequences contained D or E, accounting for 53.28%; 50 contained P, accounting for 40.98%; 32 simultaneously contained acidic residues and proline, accounting for 26.23%; and 27 began with D or E, accounting for 22.13%. This distribution was not accidental. Recent studies on umami peptides have generally suggested that umami peptides are mostly small peptides of 2 to 20 aa and are often enriched in acidic or hydrophilic residues such as E, D, Q, and N. T1R1/T1R3 also shows a high recognition tendency toward acidic and hydrophilic short peptides, and the binding process mainly relies on electrostatic interactions, hydrogen bonds, and salt bridges. Correspondingly, sequences screened in this study, such as EAAD, EITD, DQIIP, QAELIIP, QQAELIIP, YPESQQP, and YPEQP, all exhibited structural features in which acidic sites were combined with neutral or weakly hydrophobic residues. Sequences such as AEIIIP, AEILLP, LGAAVP, VSIIP, and VSIVI displayed structural characteristics in which branched-chain hydrophobic amino acids coexisted with terminal proline. This suggests that such peptides not only possess a potential basis for receptor recognition but may also differ in taste persistence and oral retention characteristics.

From the perspective of flavor reconstruction in low-alcohol lager beer, the role of these potential umami peptides is more likely to be reflected in the combined contribution of direct umami enhancement and overall body support. On the one hand, short-chain acidic peptides represented by EIP, EITD, EAAD, DQIIP, QAELIIP, QQAELIIP, YPEQP, and YPESQQP possess strong polar anchoring sites and, in theory, can more readily form stable interactions with key residues of the umami receptor, thereby serving as direct contributors to umami enhancement in low-alcohol lager beer. On the other hand, some sequences containing P, Q, S, and T may not necessarily be the core peptides with the highest umami intensity, but they may amplify and support beer-body flavor by affecting taste roundness, aftertaste persistence, and overall harmony. It should be noted that the 122 sequences do not have equal contribution potential. Sequences such as AAAIA, AAIAA, GVAA, HVAA, and VSIVI, which are dominated by hydrophobic residues but are relatively deficient in acidic sites, are more likely to participate in background taste construction or jointly influence mouthfeel balance with other components rather than act as the principal driving factors of umami. The long-standing quality shortcomings of non-alcoholic and low-alcohol beer are not limited to insufficient fruity aroma but also include thin body, weak flavor support, and incomplete overall drinking experience. The absence of ethanol further alters flavor release and mouthfeel fullness. Previous studies have shown that the addition of beer-derived umami peptides can not only increase umami scores but may also improve balance and, in some cases, reduce bitterness [16,17]. Therefore, for the sequences obtained in this study, their potential contribution to umami in low-alcohol lager beer is more likely to arise not from the independent amplification of a single peptide, but from a support network in which short-chain acidic peptides provide the main umami framework, while auxiliary peptides containing P, Q, S, and T jointly participate in body integration and mouthfeel modulation, ultimately forming umami support closer to the flavor of real beer. This result also suggests that umami reconstruction in low-alcohol lager beer is essentially closer to a peptide-synergy-driven process than to the linear addition of a single dominant sequence.

### 3.2. Analysis of the Interaction Mechanism Between Umami Peptides and Their Receptor T1R1/T1R3

**Figure 1:**
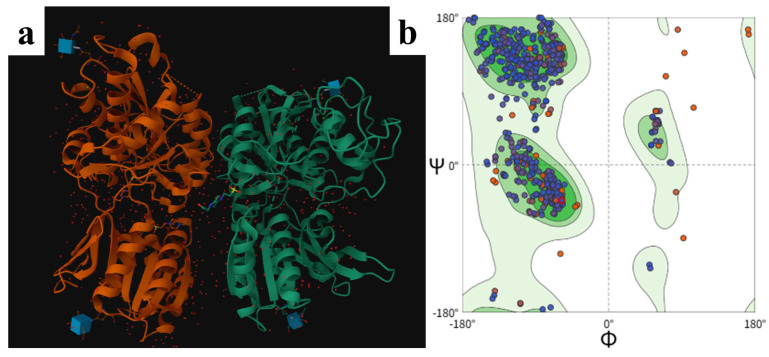
Homology model and evaluation of the umami receptor T1R1/T1R3. (**a**) Structural model of the T1R1/T1R3 heterodimer and (**b**) ramachandran plot of the model structure.

On this basis, 122 relatively representative umami peptides were further subjected to molecular docking according to their predicted umami scores and related studies. The umami receptor model used for molecular docking in this study was an extracellular recognition-domain model of T1R1/T1R3 constructed using the 1EWK structure from the RCSB database as the template. As shown in the three-dimensional structural view (Figure 1a), the model displayed a typical dimeric assembly. The two chains each formed a relatively complete folded unit. The overall conformation consisted of both α-helices and β-sheets, with a clear structural hierarchy. The two subunits were spatially adjacent, and the interfacial region formed a continuous recognition environment, thus providing a reasonable spatial basis for subsequent ligand entry and local interactions. According to the conformation shown in the figure, the main body of the receptor was compactly folded, and the secondary-structure distribution was continuous, with no obvious breaks or large-scale collapse. This indicates that the homology-modeled structure maintained good overall backbone integrity.

The model validation results showed that this receptor structure had good stereochemical rationality and docking applicability. The Ramachandran plot (Figure 1b) showed that among the non-Gly and non-Pro residues, 698 residues were located in the most favored regions, accounting for 89.1%, 82 residues were located in the additional allowed regions, accounting for 10.5%, and 3 residues were located in the generously allowed regions, accounting for 0.4%. No residues were found in the disallowed regions. These results indicate that the overall distribution of backbone dihedral angles was reasonable and that the model contained no obvious abnormal residues that violated common protein conformational rules. The basic requirements for molecular docking analysis were therefore satisfied. The PROCHECK results also showed that the model contained 899 residues in total, including 783 non-Gly and non-Pro residues, 70 Gly residues, and 36 Pro residues. This composition indicates that the statistical sample size used for Ramachandran analysis was sufficient, and that the evaluation results were reasonably representative. Further combined analysis of the ERRAT and Verify3D results showed that the model also exhibited high reliability in terms of non-bonded interaction geometry and sequence-environment compatibility. The overall quality factor from ERRAT reached 98.6286, which was far above the commonly accepted threshold for acceptable models, indicating that the pattern of non-bonded atomic interactions was highly consistent with that of high-quality experimental structures and that the model contained few internal geometric conflicts. The Verify3D results showed that 89.74% of the residues had an average 3D-1D score of not less than 0.1, and the model passed the criterion requiring at least 80.0% of residues to reach this threshold. This indicates that the local three-dimensional environments of most amino acid residues matched their primary-sequence properties well, and that no large-scale environmental mismatch occurred in the model.

On this basis, molecular docking was performed, and the specific docking results are shown in Table 2. Statistical analysis of the 117 successfully docked umami peptides showed a clear enrichment of medium-short peptides in terms of peptide-length distribution. Among them, tetrapeptides were the most abundant, with 47 sequences accounting for 40.17%. Pentapeptides numbered 42, accounting for 35.90%. Tripeptides and hexapeptides numbered 12 and 11, accounting for 10.26% and 9.40%, respectively. Only 4 dipeptides were detected, accounting for 3.42%, and only 1 heptapeptide was detected, accounting for 0.85%. These results indicate that the successfully docked peptides screened in this study were mainly concentrated in the length range of 4 to 5 amino acids, suggesting that medium-short peptides were more likely to enter the receptor recognition range and form stable conformations during the early virtual screening process.

From the overall distribution of docking scores, all 117 umami peptides showed negative docking scores, ranging from −7.80 to −1.71 kcal/mol, with a mean of −5.22 and a median of −5.14. The interquartile range was between −5.75 and −4.80. According to the score intervals, 4 peptides had docking scores ≤−7.00, accounting for 3.42%. A total of 21 peptides had scores from −7.00 to −6.00, accounting for 17.95%. A total of 43 peptides had scores from −6.00 to −5.00, accounting for 36.75%. A total of 37 peptides had scores from −5.00 to −4.00, accounting for 31.62%. Only 12 peptides had scores higher than −4.00, accounting for 10.26%. In other words, 68 peptides had scores lower than −5.00, accounting for 58.12%, and 25 peptides had scores lower than −6.00, accounting for 21.37%. This indicates that most candidate peptides were able to form relatively stable predicted binding with the receptor.

Further analysis by peptide length showed that tetrapeptides had the lowest mean docking score, at −5.36, followed by hexapeptides at −5.22 and pentapeptides at −5.14, whereas dipeptides showed a mean score of only −4.43, indicating relatively weak overall binding performance. However, in terms of the best individual sequences, the top-ranked peptides were not concentrated in a single peptide-length category. The highest-scoring sequence, CTGAA, was a pentapeptide. IDQILG, ranked third, was a hexapeptide. QRQ, ranked seventh, was a tripeptide. These results indicate that peptide length affects receptor entry and spatial matching, but is not the only determinant of binding ability. Amino acid composition, polarity distribution, and spatial arrangement more directly influence binding stability. In terms of scoring-item differences, the mean values of glide ecoul, glide hbond, glide evdw, and glide energy for the top 10 high-scoring peptides were −22.28, −1.28, −38.17, and −60.45, respectively. These values were all markedly lower than those of the bottom 10 low-scoring peptides, which were −11.73, −0.58, −33.46, and −45.19, respectively. This suggests that the advantage of high-scoring peptides mainly arose from stronger electrostatic matching, more sufficient hydrogen-bonding interactions, and more favorable van der Waals contacts.

On this basis, CTGAA, IDQILG, KDTHP, QRQ, and EITGR were finally selected for subsequent analysis of their specific interaction mechanisms with the umami receptor, and this selection showed strong representativeness and rationality. In terms of score performance, the docking scores of these five peptides were −7.80, −7.53, −6.90, −6.74, and −6.61, respectively. Among all 117 sequences, they ranked first, third, fifth, seventh, and ninth, respectively, and all were within the top 10. CTGAA was the highest-scoring sequence in the entire dataset. IDQILG was the highest-scoring hexapeptide. QRQ was the highest-scoring tripeptide. These findings indicate that the five peptides not only ranked highly overall but were also representative within different peptide-length categories. Further analysis showed that CTGAA, as the highest-scoring pentapeptide, was suitable as a representative strong-binding sequence for analyzing the typical receptor recognition mode of high-scoring umami peptides. IDQILG showed a glide energy of −68.29 and a glide evdw value of −50.12, both of which were notable among the five candidate peptides, indicating an advantage in pocket filling and hydrophobic contact. KDTHP showed a glide model as low as −97.96, the best among the five candidate peptides, suggesting strong conformational stability after receptor docking and making it suitable for observing conformational maintenance and key-site locking behavior. Although QRQ was only a tripeptide, its docking score still reached −6.74, indicating that short peptides also possess strong receptor recognition potential and are therefore useful for comparing binding differences under different chain-length conditions. EITGR showed a score of −6.61, which remained stable within the top 10. At the same time, its sequence contains the acidic residue E, the basic residue R, and the hydrophobic residue I. Its physicochemical features are therefore relatively rich, which is favorable for analyzing receptor recognition mechanisms from the perspective of synergistic multiple interactions. More importantly, these five peptides covered three peptide-length categories, namely tripeptides, pentapeptides, and hexapeptides. In terms of amino acid composition, they also included acidic, basic, neutral, sulfur-containing, and hydrophobic features. This effectively avoided overconcentration of the subsequent mechanistic analysis on a single peptide length or a single sequence type. Combined with previous studies showing the importance of acidic residues such as D and E, as well as hydrogen bonding and electrostatic interactions, in T1R1/T1R3 recognition, these five peptides possessed high score advantages, strong category representativeness, and good structural diversity, and were therefore suitable as key targets for subsequent analysis of molecular interaction mechanisms.

Compared with known umami peptides reported in beer and other fermented foods, the 5 selected peptides in this study showed both shared and distinctive structural features. Many reported umami peptides contain acidic residues such as D or E, and this feature is generally considered favorable for T1R1/T1R3 recognition [17]. IDQILG, KDTHP, and EITGR were consistent with this tendency, since they contained D or E and showed strong predicted receptor binding. However, CTGAA and QRQ did not fully follow this classical acidic residue pattern, yet they still ranked among the top candidates. This result suggests that umami activity in NAB may not depend on a single residue type. Chain length, residue order, polarity distribution, hydrogen bonding capacity, and pocket fitting may act together. Compared with previously reported beer umami peptides such as EESY, IEVVD, EIVDV, and IGVND [53], the peptides identified here were also concentrated in the short to medium length range, but covered more diverse residue compositions. This diversity may be useful for explaining why different umami peptides can produce similar taste tendencies through different receptor interaction modes [54]. Therefore, the selected peptides were not only comparable with known umami peptides in length and receptor binding potential, but also provided new sequence examples for understanding peptide-mediated umami contribution in NAB.

**Table 2:** Docking binding energies of 117 umami peptides with the receptor T1R1/T1R3.

Peptide	Docking Score	Glide Ecoul	Glide Emodel	Glide Energy	Glide Esite	Glide Evdw	Glide Gscore	Glide Hbond	Glide Ligand Efficiency	Glide Ligand Efficiency ln	Glide Ligand Efficiency sa	Glide Lipo	Glide Rewards
CTGAA	−7.80	−23.04	−77.86	−57.10	−0.06	−34.06	−7.92	−1.94	−0.28	−1.80	−0.85	−0.89	−1.44
ARNK	−7.62	−26.81	−99.36	−63.39	−0.14	−36.59	−7.74	−1.71	−0.22	−1.68	−0.73	−0.79	−0.72
IDQILG	−7.53	−18.17	−83.94	−68.29	0.00	−50.12	−8.35	−1.16	−0.16	−1.56	−0.59	−2.05	−0.79
NAAAP	−7.04	−19.36	−75.26	−55.91	−0.03	−36.55	−7.22	−1.47	−0.23	−1.59	−0.71	−1.26	−0.96
KDTHP	−6.90	−24.23	−97.96	−67.91	−0.08	−43.68	−8.21	−0.74	−0.16	−1.46	−0.57	−1.39	−0.82
DQPRT	−6.80	−19.66	−87.82	−61.42	−0.03	−41.76	−6.97	−0.76	−0.16	−1.43	−0.55	−1.49	−0.63
QRQ	−6.74	−21.05	−81.54	−55.12	−0.17	−34.07	−7.23	−1.91	−0.22	−1.53	−0.70	−0.61	−1.28
ARIP	−6.73	−20.99	−85.85	−55.39	−0.17	−34.40	−6.85	−0.76	−0.21	−1.51	−0.67	−1.59	−0.71
EITGR	−6.61	−22.78	−92.51	−67.19	−0.08	−44.41	−7.18	−1.11	−0.17	−1.41	−0.57	−1.08	−0.50
KSVS	−6.59	−26.73	−81.22	−52.75	−0.15	−26.03	−6.84	−1.23	−0.23	−1.51	−0.70	−0.79	−0.95
EAAYPS	−6.55	−14.00	−80.79	−61.71	−0.05	−47.72	−6.82	−0.82	−0.15	−1.36	−0.52	−2.03	−0.30
RKHE	−6.54	−33.10	−105.07	−67.87	−0.05	−34.77	−7.46	−0.93	−0.16	−1.40	−0.56	−0.45	−1.13
SGICAAD	−6.53	−18.77	−88.62	−68.72	−0.06	−49.95	−7.03	−0.76	−0.15	−1.37	−0.53	−1.34	−0.57
PSCT	−6.50	−16.02	−69.77	−49.08	−0.13	−33.06	−6.52	−1.44	−0.24	−1.51	−0.72	−0.90	−1.30
DIAR	−6.49	−22.13	−78.48	−54.83	−0.13	−32.70	−6.65	−0.72	−0.20	−1.44	−0.63	−0.91	−0.95
INNAA	−6.47	−18.13	−87.42	−65.38	−0.04	−47.25	−7.30	−1.48	−0.18	−1.42	−0.60	−1.23	−0.68
TTHHP	−6.39	−21.58	−94.20	−66.40	−0.23	−44.81	−7.21	−0.90	−0.15	−1.35	−0.53	−1.10	−0.28
TTCAA	−6.25	−24.08	−80.40	−60.19	−0.13	−36.12	−6.57	−0.84	−0.20	−1.41	−0.63	−0.72	−0.85
TVDPST	−6.16	−15.80	−73.79	−63.67	−0.16	−47.87	−6.75	−1.00	−0.14	−1.29	−0.50	−1.25	−0.44
PEHA	−6.11	−20.15	−77.77	−54.68	−0.18	−34.53	−6.37	−0.68	−0.19	−1.37	−0.61	−0.95	−0.89
PGDPAR	−6.04	−10.43	−81.53	−53.73	−0.09	−43.30	−6.06	−0.70	−0.14	−1.27	−0.49	−0.67	−1.53
DKDP	−6.03	−18.36	−76.52	−51.66	−0.13	−33.30	−6.19	−0.91	−0.18	−1.34	−0.59	−0.84	−1.07
AADF	−6.02	−19.74	−71.67	−48.24	−0.13	−28.50	−6.15	−1.19	−0.20	−1.37	−0.62	−0.73	−0.83
NIN	−6.02	−15.12	−67.00	−48.74	0.00	−33.62	−6.23	−1.26	−0.24	−1.43	−0.70	−0.52	−1.84
AACH	−6.00	−23.03	−84.98	−61.35	0.00	−38.32	−6.69	−0.89	−0.22	−1.40	−0.67	−1.33	−0.32
MIEIP	−5.93	−10.74	−56.59	−43.49	−0.01	−32.75	−6.37	−1.29	−0.14	−1.26	−0.50	−2.08	−0.61
NIT	−5.87	−18.15	−58.03	−47.91	−0.01	−29.76	−6.08	−1.39	−0.24	−1.40	−0.71	−0.13	−1.78
MKD	−5.79	−23.36	−76.26	−50.29	−0.11	−26.92	−6.22	−0.35	−0.22	−1.36	−0.66	−1.13	−1.31
HPST	−5.78	−21.39	−72.51	−55.95	−0.14	−34.57	−6.14	−0.84	−0.19	−1.30	−0.59	−0.89	−0.47
ESPAA	−5.75	−15.12	−73.22	−53.28	−0.02	−38.15	−6.06	−0.65	−0.17	−1.28	−0.56	−1.41	−1.07
AVSH	−5.73	−24.94	−75.79	−57.35	0.00	−32.41	−6.26	−1.50	−0.20	−1.31	−0.61	−0.94	0.39
AVSI	−5.72	−16.09	−61.91	−48.11	−0.04	−32.02	−6.78	−1.46	−0.21	−1.33	−0.64	−1.62	−0.94
QQAE	−5.67	−25.38	−77.01	−57.63	−0.14	−32.25	−6.01	−1.29	−0.17	−1.26	−0.55	−0.15	−0.36
AHAR	−5.66	−20.92	−80.03	−53.41	−0.15	−32.50	−6.45	−0.77	−0.18	−1.27	−0.56	−0.40	−1.33
TCQA	−5.66	−21.01	−75.70	−53.87	−0.11	−32.86	−5.99	−1.07	−0.20	−1.31	−0.61	−0.50	−0.96
AEIIIP	−5.63	−13.88	−58.01	−53.46	−0.04	−39.57	−6.62	−0.71	−0.12	−1.17	−0.44	−1.92	−0.63
PPEIP	−5.58	−7.41	−57.50	−51.60	−0.04	−44.19	−5.59	−0.35	−0.14	−1.20	−0.48	−1.79	−0.90
VSIE	−5.57	−16.41	−70.60	−52.51	−0.17	−36.09	−5.78	−0.93	−0.18	−1.26	−0.56	−1.06	−0.67
ARGGK	−5.56	−23.03	−86.18	−56.89	−0.09	−33.86	−5.69	−0.65	−0.16	−1.23	−0.53	−0.89	−0.52
KVSS	−5.56	−24.11	−70.61	−51.73	−0.03	−27.62	−5.79	−0.89	−0.19	−1.27	−0.59	−0.54	−0.90
CADHV	−5.54	−17.84	−77.69	−59.60	−0.20	−41.76	−6.70	−1.13	−0.15	−1.20	−0.50	−1.36	−0.30
GVAA	−5.50	−14.83	−61.46	−43.59	−0.04	−28.76	−5.63	−0.84	−0.25	−1.35	−0.70	−0.78	−1.80
PARSI	−5.50	−22.24	−82.37	−58.86	−0.25	−36.62	−5.51	−0.59	−0.14	−1.19	−0.49	−0.74	−0.03
EKDGA	−5.47	−16.73	−66.42	−53.02	−0.08	−36.29	−5.75	−0.78	−0.15	−1.19	−0.50	−1.13	−0.82
AEILLP	−5.46	−9.06	−73.08	−59.41	−0.01	−50.35	−5.58	−0.01	−0.12	−1.13	−0.42	−1.66	−0.76
ARPC	−5.38	−18.74	−78.35	−54.59	−0.13	−35.85	−6.36	−1.03	−0.18	−1.22	−0.56	−1.07	−0.84
AATM	−5.36	−17.46	−67.23	−48.58	−0.04	−31.12	−5.48	−0.46	−0.21	−1.26	−0.61	−1.10	−1.10
AAAVP	−5.35	−17.04	−46.56	−40.15	−0.16	−23.11	−5.35	−0.94	−0.18	−1.22	−0.55	−1.23	−0.41
VDV	−5.35	−13.08	−56.07	−38.63	−0.10	−25.55	−5.53	−0.64	−0.23	−1.29	−0.66	−1.12	−1.63
ISPSK	−5.34	−21.53	−74.72	−57.07	−0.09	−35.54	−6.16	−0.91	−0.14	−1.16	−0.48	−1.13	−0.27
IHAD	−5.28	−16.08	−74.57	−57.20	−0.01	−41.12	−6.31	−0.74	−0.16	−1.18	−0.52	−1.17	−1.10
IDQP	−5.26	−20.94	−59.03	−47.94	−0.25	−27.00	−5.43	−0.90	−0.16	−1.17	−0.51	−0.62	−0.26
DC	−5.24	−16.31	−47.61	−32.73	−0.02	−16.42	−5.42	−0.80	−0.35	−1.41	−0.86	−0.39	−2.50
ASCH	−5.23	−14.95	−69.54	−49.45	−0.10	−34.50	−5.78	−1.02	−0.19	−1.21	−0.57	−0.85	−1.09
EAATQ	−5.18	−19.94	−74.39	−56.93	−0.13	−36.99	−5.45	−0.96	−0.14	−1.13	−0.48	−0.45	−0.38
QVSTE	−5.18	−16.80	−74.32	−55.06	−0.07	−38.25	−5.52	−0.78	−0.13	−1.11	−0.45	−0.74	−0.61
NINI	−5.15	−12.56	−62.90	−51.18	−0.08	−38.62	−5.33	−0.91	−0.16	−1.15	−0.50	−1.00	−0.71
EYST	−5.15	−18.99	−66.31	−52.74	−0.01	−33.75	−5.70	−1.32	−0.15	−1.13	−0.48	−0.66	−0.41
EVSH	−5.14	−24.86	−79.87	−56.17	−0.21	−31.31	−6.99	−1.80	−0.16	−1.14	−0.50	−0.45	−0.42
ACHA	−5.14	−15.32	−71.93	−50.20	−0.14	−34.88	−5.52	−0.81	−0.19	−1.20	−0.57	−0.52	−1.23
QSPAA	−5.11	−13.40	−63.99	−51.21	−0.04	−37.81	−5.55	−0.96	−0.15	−1.14	−0.50	−0.96	−0.96
SVSD	−5.10	−20.06	−63.44	−53.57	−0.04	−33.51	−5.46	−1.75	−0.18	−1.18	−0.55	−0.90	0.47
TYTV	−5.09	−19.00	−66.40	−54.89	−0.03	−35.88	−5.62	−0.77	−0.15	−1.12	−0.48	−0.79	−0.52
ESYP	−5.07	−16.93	−68.43	−51.46	−0.05	−34.53	−5.38	−0.56	−0.14	−1.11	−0.47	−1.42	−0.17
TFD	−5.06	−12.43	−54.15	−37.43	−0.10	−25.00	−5.40	−0.88	−0.19	−1.18	−0.56	−1.65	−0.86
AVSMPP	−5.05	−12.69	−49.77	−41.06	−0.02	−28.37	−5.17	−0.53	−0.12	−1.07	−0.42	−1.26	−0.80
EITD	−5.03	−21.14	−59.57	−51.65	−0.03	−30.51	−5.55	−0.95	−0.15	−1.12	−0.49	−0.60	−0.54
AATAR	−5.02	−19.75	−74.90	−53.65	−0.26	−33.90	−5.15	−0.55	−0.15	−1.11	−0.48	−0.72	−0.41
ASTE	−4.97	−21.47	−64.44	−49.15	−0.04	−27.68	−5.11	−1.56	−0.18	−1.15	−0.54	−0.63	0.30
IDMIP	−4.96	−10.02	−65.67	−52.46	−0.04	−42.45	−5.13	−0.32	−0.12	−1.06	−0.42	−1.51	−0.51
CTRK	−4.93	−23.27	−72.47	−60.44	−0.11	−37.17	−5.05	−0.18	−0.15	−1.09	−0.47	−0.78	−0.14
VSAAH	−4.93	−19.40	−79.24	−57.02	−0.08	−37.62	−5.53	−0.70	−0.15	−1.09	−0.47	−0.57	−0.61
AAAIA	−4.93	−15.88	−64.36	−48.60	−0.13	−32.72	−5.05	−0.68	−0.17	−1.13	−0.52	−0.78	−0.82
ARAAC	−4.92	−17.90	−72.84	−52.97	−0.22	−35.07	−5.04	−0.37	−0.15	−1.09	−0.48	−0.99	−0.46
DQIIP	−4.91	−9.13	−69.55	−57.58	−0.03	−48.45	−5.07	−0.32	−0.12	−1.04	−0.41	−1.20	−0.61
GTAA	−4.91	−15.92	−61.86	−43.79	−0.01	−27.87	−5.07	−0.66	−0.22	−1.20	−0.62	−0.54	−1.73
HVAA	−4.91	−21.81	−67.67	−50.23	−0.13	−28.42	−5.34	−0.54	−0.18	−1.13	−0.53	−0.53	−0.69
EAAD	−4.91	−16.54	−60.41	−44.81	−0.15	−28.27	−5.20	−0.69	−0.18	−1.13	−0.53	−0.73	−1.19
DIVSAR	−4.90	−17.80	−76.91	−62.18	−0.10	−44.38	−5.74	−0.49	−0.11	−1.01	−0.38	−1.27	0.32
VSTEP	−4.90	−14.11	−67.92	−49.19	−0.04	−35.07	−5.09	−0.60	−0.13	−1.06	−0.44	−1.33	−0.35
AATP	−4.87	−10.61	−58.28	−41.72	−0.01	−31.11	−4.99	−0.52	−0.19	−1.16	−0.57	−1.08	−1.43
PE	−4.87	−14.04	−44.16	−31.45	−0.04	−17.41	−4.89	−0.34	−0.29	−1.27	−0.74	−0.70	−2.01
VPSQP	−4.86	−16.18	−66.72	−55.34	−0.13	−39.17	−4.93	−0.64	−0.13	−1.05	−0.44	−0.51	−0.24
EIP	−4.84	−15.48	−55.20	−42.31	−0.05	−26.83	−5.13	−0.38	−0.19	−1.15	−0.57	−0.87	−1.38
AACT	−4.84	−16.66	−60.43	−45.92	−0.10	−29.27	−5.00	−0.70	−0.20	−1.16	−0.58	−0.65	−1.04
MPSE	−4.81	−13.41	−54.40	−44.66	−0.02	−31.25	−5.02	−0.74	−0.16	−1.09	−0.49	−0.95	−0.96
KKDI	−4.80	−23.74	−70.04	−55.77	−0.10	−32.03	−5.46	−0.40	−0.14	−1.05	−0.45	−1.03	−0.15
VSIIP	−4.80	−12.07	−64.89	−48.06	−0.04	−35.99	−5.00	−0.76	−0.13	−1.04	−0.43	−1.47	−0.16
QPAAP	−4.77	−13.01	−67.16	−56.79	−0.02	−43.78	−5.55	−0.41	−0.14	−1.05	−0.45	−1.29	−0.75
EIKYQ	−4.77	−17.04	−60.39	−51.23	−0.13	−34.19	−5.06	−1.34	−0.10	−0.98	−0.36	−1.60	1.43
EII	−4.71	−15.34	−54.83	−41.99	−0.07	−26.65	−5.00	−0.58	−0.18	−1.11	−0.54	−0.99	−1.11
DP	−4.68	−9.66	−42.35	−30.26	0.00	−20.60	−4.79	−0.38	−0.29	−1.24	−0.74	−0.51	−2.39
TVSP	−4.66	−15.55	−56.25	−44.82	−0.03	−29.28	−4.94	−0.80	−0.17	−1.08	−0.51	−0.79	−0.73
AGAAT	−4.64	−17.27	−64.00	−51.66	−0.01	−34.39	−4.78	−0.32	−0.17	−1.08	−0.52	−0.63	−1.05
YPEQP	−4.61	−4.81	−61.44	−48.16	−0.08	−43.35	−4.97	−0.24	−0.10	−0.96	−0.36	−1.77	−0.70
EAAHP	−4.60	−10.13	−66.70	−53.54	−0.14	−43.41	−5.40	−0.78	−0.12	−1.00	−0.41	−1.18	−0.67
MID	−4.48	−11.93	−52.49	−38.21	−0.09	−26.27	−4.91	−0.71	−0.18	−1.06	−0.52	−1.19	−1.19
GAAVE	−4.45	−11.64	−63.92	−50.80	−0.05	−39.17	−4.58	−0.25	−0.14	−1.00	−0.45	−0.44	−1.45
AAGGAY	−4.44	−13.65	−71.54	−56.34	−0.14	−42.69	−4.56	−0.28	−0.12	−0.97	−0.41	−1.09	−0.14
EIA	−4.38	−12.88	−48.49	−38.67	−0.03	−25.79	−4.67	−0.49	−0.19	−1.06	−0.54	−0.78	−1.70
ASI	−4.37	−12.74	−41.71	−34.22	−0.01	−21.48	−4.37	−0.79	−0.22	−1.09	−0.59	−0.40	−2.35
IGAR	−4.37	−26.93	−62.12	−47.96	−0.27	−21.03	−4.54	−0.46	−0.15	−1.00	−0.46	−0.46	−0.01
DAAP	−4.35	−14.98	−49.88	−42.08	−0.04	−27.10	−4.52	−0.42	−0.17	−1.02	−0.50	−0.52	−1.20
DVSP	−4.31	−11.37	−46.74	−42.34	−0.06	−30.97	−4.48	−0.32	−0.15	−0.99	−0.46	−0.73	−1.25
VTPSK	−4.23	−16.45	−67.24	−53.52	−0.06	−37.07	−4.43	−0.28	−0.11	−0.92	−0.38	−0.74	−0.19
ARIQL	−3.86	−20.03	−74.67	−57.17	−0.34	−37.15	−3.98	−0.76	−0.09	−0.81	−0.32	−0.41	1.34
AAIAA	−3.84	−14.34	−58.30	−49.12	−0.16	−34.77	−3.97	−0.68	−0.13	−0.88	−0.41	−1.19	0.58
YPVD	−3.81	−9.92	−58.18	−44.65	−0.06	−34.73	−4.21	−0.35	−0.11	−0.84	−0.36	−1.09	−0.41
QACA	−3.70	−14.24	−59.37	−47.86	−0.25	−33.61	−4.04	−1.05	−0.14	−0.87	−0.42	−0.50	0.05
ADASV	−3.70	−20.27	−61.79	−52.20	−0.12	−31.93	−3.84	−0.79	−0.12	−0.83	−0.37	−0.25	0.73
IAEIP	−3.49	−7.77	−52.74	−46.54	−0.01	−38.78	−3.65	−0.07	−0.09	−0.75	−0.31	−0.61	−0.91
GSK	−3.18	−19.17	−50.58	−42.75	0.00	−23.58	−4.05	−0.88	−0.16	−0.79	−0.43	−0.89	−2.11
VSIVI	−3.14	−3.74	−48.13	−41.90	−0.02	−38.16	−3.33	−0.44	−0.08	−0.68	−0.28	−1.22	−0.28
DSTYQP	−3.12	−6.22	−50.06	−47.56	−0.20	−41.34	−3.30	0.00	−0.06	−0.64	−0.23	−1.82	0.99
EI	−2.92	−11.86	−38.17	−33.40	−0.01	−21.54	−3.52	−1.12	−0.16	−0.75	−0.42	−0.67	−2.03
LGAAVP	−2.50	−10.09	−57.02	−51.80	−0.06	−41.71	−2.71	−0.20	−0.07	−0.54	−0.23	−1.24	1.27
AAKKK	−1.71	−14.00	−39.71	−43.26	−0.11	−29.26	−2.69	−0.85	−0.05	−0.37	−0.15	−0.80	1.66

Note: All values are expressed in kcal/mol. Docking score indicates the overall docking score. Glide gscore indicates the GlideScore. Differences may occur between the two when an Epik state penalty is introduced during scoring. Glide emodel indicates the conformational selection score. Glide ecoul and glide evdw indicate the Coulomb interaction energy and van der Waals interaction energy, respectively, whereas glide energy indicates the corrected interaction energy. Glide esite, glide hbond, glide lipo, and glide rewards indicate the active-site polar interaction term, hydrogen-bonding term, lipophilic interaction term, and other reward or penalty terms, respectively. Glide ligand efficiency, glide ligand efficiency ln, and glide ligand efficiency sa indicate ligand-efficiency indices corrected for molecular size. The renderings of GlideScore, Emodel, and related scoring terms are consistent with Schrödinger’s docking documentation.

### 3.3. Molecular Mechanism Analysis of Five Umami Peptides and Their Receptor Proteins

**Figure 2:**
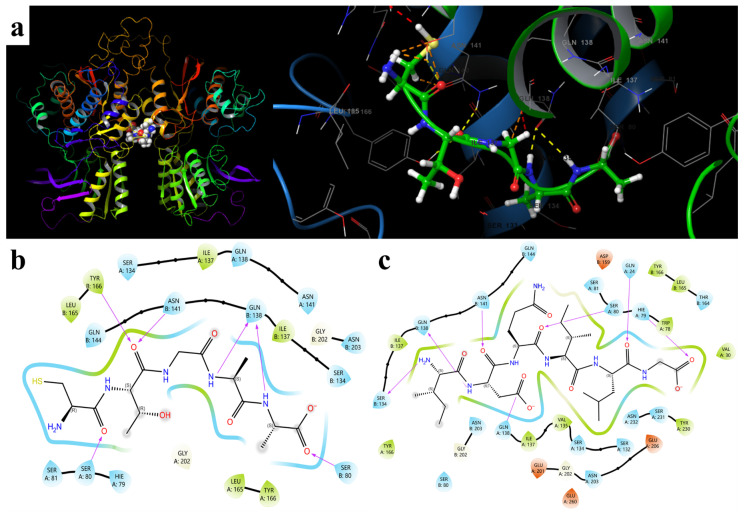

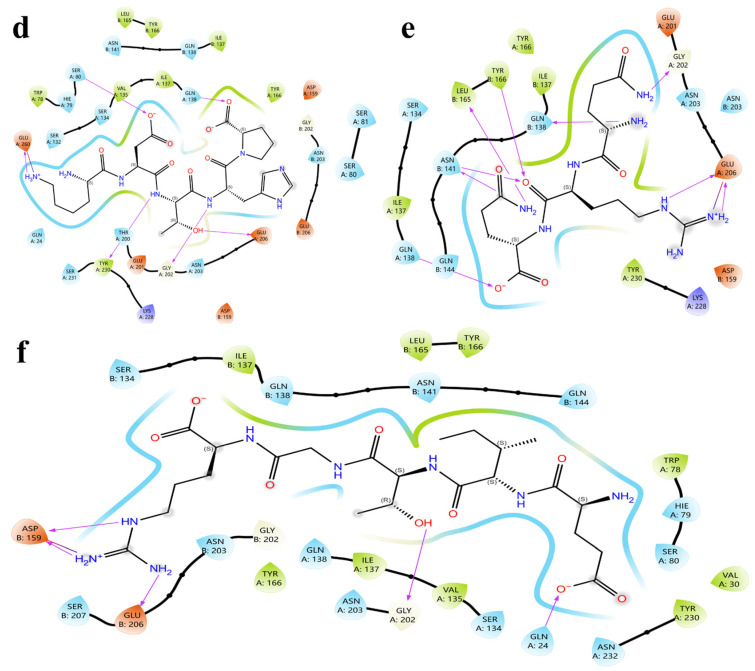
Schematic representation of molecular docking. The left panel shows the overall view, and the right panel shows a close-up view of the intermolecular interactions (**a**). Hydrogen bonds are shown in blue, halogen bonds in purple, salt bridges in red, aromatic hydrogen bonds in cyan, π–π stacking interactions in blue, and π–cation interactions in green. The 2D binding mode diagrams of CTGAA, IDQILG, KDTHP, QRQ, and EITGR with T1R1/T1R3, obtained from docking, are shown in panels (**b**,**c**,**d**,**e**,**f**), respectively.

The recognition of the umami peptide CTGAA by the umami receptor T1R1/T1R3 mainly occurred in the extracellular Venus flytrap binding region of the receptor, which is the key interface for umami ligand entry, positioning, and induction of conformational changes (Figure 2a). The two-dimensional docking diagram showed that CTGAA mainly formed multipoint anchoring interactions with the receptor through backbone carbonyl and amide groups (Figure 2b). Ser A80, Tyr B166, Asn B141, Gln B138, and Ser B80 were the key interacting residues, and a total of six hydrogen bonds were formed. Gln B138 simultaneously interacted with two amide hydrogens in the middle-to-late segment, thereby conferring continuous stabilization to the middle region of the peptide chain. The interactions of Ser A80 with the N-terminal carbonyl group and of Ser B80 with the terminal carboxyl group restricted conformational fluctuation at both ends. At the same time, Ser A134, Gln A138, Asn A141, and Gln B144 formed a hydrophilic microenvironment around the binding cavity, whereas Ile A137, Ile B137, Leu A165, Leu B165, Tyr A166, and Tyr B166 provided local hydrophobic burial and spatial support. These results indicate that recognition of CTGAA did not rely on a single strong bond, but instead stabilized the T1R1/T1R3 complex through multipoint hydrogen-bond fixation, polar matching, and hydrophobic cooperation, thereby favoring receptor conformational response and umami signal transduction.

As shown in Figure 2c, the peptide formed seven major anchoring sites within the receptor pocket. Ser B134 interacted with the N-terminal amino region of the peptide. Gln B138 and Asn B141 acted on two carbonyl oxygen atoms in the front segment. Ser A80 stabilized the cyclic amide carbonyl group in the middle region. Gln A24 recognized the backbone carbonyl group. His A79 interacted with the terminal carboxyl oxygen atom. Gln A138 formed an additional polar pairing with the side-chain carboxylic acid group. These results indicate that binding of the peptide did not depend on a single strong interaction, but achieved spatial restraint through a multipoint hydrogen-bonding network composed of front-segment entry, middle-segment locking, and terminal fixation. At the same time, neighboring residues such as Ile A137, Val A135, Tyr B166, Leu B165, and Trp A78 jointly formed a local hydrophobic microenvironment, which helped reduce peptide-chain fluctuation and improve complex stability. Overall, this umami peptide mainly stabilized the T1R1/T1R3 complex through multipoint hydrogen-bond support, directional recognition of polar groups, and cooperative hydrophobic interactions. This binding mode favored maintenance of a receptor conformation more conducive to signal transduction and provided a structural basis for the molecular response of umami perception.

The key anchoring sites of this peptide mainly included Glu A260, Val A135, Gln A138, Thr A200, Gly A202, and Glu A206. A clear electrostatic attraction was formed between the protonated amino group at the N terminus of the peptide chain and Glu A260, which determined the initial orientation of the ligand after entry into the pocket. The carboxyl group of the acidic side chain in the middle region was recognized by the backbone site of Val A135 and the polar site of Gln A138, thereby providing dual fixation of the peptide chain in the upper part of the binding cavity. The internal amide NH of the peptide chain formed a hydrogen bond with Thr A200, which further restricted backbone fluctuation. Polar contacts were also established near the side-chain hydroxyl group with Gly A202 and Glu A206, making the conformation of the middle-to-late segment more compact. At the same time, neighboring residues such as Ile A137, Ile B137, Leu B165, Tyr B166, Trp A78, and Ser A80 formed a local microenvironment with alternating hydrophobic and hydrophilic distribution around the ligand, which helped reduce peptide-chain flexibility and improve complex stability (Figure 2d).

Stable binding of umami peptides to T1R1/T1R3 is usually dominated by hydrogen bonding and electrostatic interactions, whereas hydrophobic residues more often serve local burial and conformational buffering functions. The two-dimensional interaction diagram showed that Gln B138 performed directional recognition of the upper amino group, Gly A202 stabilized the side-chain amide carbonyl group, Asn B141 recognized the carbonyl oxygen in the middle segment, and Gln B144 formed a polar pairing with the terminal carboxylate, indicating that the peptide formed a continuous multipoint anchoring network in the binding cavity (Figure 2e). Two concentrated interaction sites were observed between the guanidinium group at the right end and Glu A206, suggesting that the peptide-chain terminus was strongly fixed through the combined effect of electrostatic attraction and hydrogen bonding. This site was likely the core anchor for maintenance of complex stability. At the same time, Ile B137, Leu B165, and Tyr B166 were distributed around the middle-to-upper region of the peptide chain and provided a certain degree of hydrophobic burial and spatial restriction, allowing polar recognition and hydrophobic support to act cooperatively.

Recognition of this umami peptide by the umami receptor T1R1/T1R3 mainly occurred within the ligand-binding cavity of the extracellular Venus flytrap domain (Figure 2f). The key interacting residues of this peptide mainly included Asp B159, Glu B206, Leu B165, Tyr B166, Gly A202, and Gln A24. The positively charged guanidinium group at the left end of the peptide chain simultaneously pointed toward Asp B159 and was adjacent to Glu B206, indicating that the receptor formed an evident negatively charged recognition center in this region. This interaction was decisive for initial peptide adsorption and terminal fixation. The amide carbonyl group in the middle region was adjacent to Leu B165 and Tyr B166, suggesting that this region exerted a certain spatial confinement effect in addition to polar recognition, thereby helping compress peptide-chain freedom and stabilize the local conformation. The hydroxyl group in the middle-to-late segment contacted Gly A202, whereas the terminal carboxylate was directionally recognized by Gln A24. These results indicate that the receptor adopted a layered recognition mode for this peptide that combined front-end charge adsorption, middle-segment conformational restraint, and terminal polar locking.

Comprehensive analysis of the docking results shown in Figure 2a–f indicated that recognition of umami peptides by the umami receptor T1R1/T1R3 mainly occurred within the ligand-binding cavity of the extracellular Venus flytrap domain. This region is the core interface for ligand entry, positioning, and induction of conformational response, and ligand binding can further affect pocket opening and closing as well as subsequent signal transduction. In terms of the specific interaction mode, umami peptides did not rely on a single high-intensity bond for binding, but instead established stable complexes through multipoint hydrogen bonding, electrostatic attraction, and local hydrophobic burial. Among these interactions, polar residues such as Ser, Gln, Asn, Gly, Thr, and His mainly recognize backbone carbonyl groups, amide hydrogens, carboxyl groups, and hydroxyl groups, thereby forming a continuous hydrogen-bonding network that enabled a layered recognition mode consisting of front-segment guidance, middle-segment locking, and terminal fixation within the binding cavity. At the same time, acidic residues such as Glu and Asp formed marked electrostatic adsorption with positively charged amino or guanidinium groups, and this effect was especially prominent in initial peptide orientation and terminal reinforcement, indicating that the receptor contained recognition centers highly sensitive to charge distribution. In contrast, hydrophobic or aromatic residues such as Ile, Leu, Val, Tyr, and Trp did not directly dominate polar recognition, but reduced peptide-chain flexibility and compressed the range of free fluctuation through spatial confinement, hydrophobic burial, and conformational buffering, thereby enhancing ligand residence stability within the pocket. These results indicate that the interaction between umami peptides and T1R1/T1R3 is essentially a cooperative recognition process dominated by hydrogen bonding and electrostatic interactions, with hydrophobic support as an auxiliary factor. This mechanism not only ensures that umami peptides with different structures can achieve effective anchoring within the receptor cavity, but also provides a structural basis for maintenance of a receptor conformation more favorable for umami signal transduction.

### 3.4. Analysis of the MM-GBSA Binding Energy Results of Five Umami Peptides

Comparison of the MM-GBSA results of the five umami peptides showed clear differences among the sequences in total binding free energy, no-strain binding free energy, and the contributions of individual energy terms (Table 3).

In terms of total binding free energy, the MMGBSA dG Bind values of the five peptides were −45.21 kcal/mol for CTGAA, −39.47 kcal/mol for QRQ, −28.83 kcal/mol for IDQILG, −28.78 kcal/mol for EITGR, and −14.28 kcal/mol for KDTHP. These results indicate that CTGAA showed the most stable overall binding, followed by QRQ, whereas KDTHP showed the weakest net binding advantage. When the no-strain binding free energy was considered, the ranking changed. The values were −72.14 kcal/mol for EITGR, −65.74 kcal/mol for CTGAA, −64.06 kcal/mol for QRQ, −60.47 kcal/mol for IDQILG, and −51.40 kcal/mol for KDTHP. This indicates that EITGR had the strongest intrinsic binding potential under ideal contact conditions, but its actual total binding energy did not show the same advantage, suggesting that this peptide required a higher conformational adjustment cost to enter the receptor pocket and form a stable complex. When the difference between dG Bind and dG Bind (NS) was regarded as the strain cost, the strain costs of CTGAA, QRQ, IDQILG, KDTHP, and EITGR were 20.53, 24.59, 31.64, 37.12, and 43.36 kcal/mol, respectively. CTGAA showed the lowest value, whereas EITGR showed the highest. This result indicates that the final binding performance was determined not only by the strength of local interactions, but also by whether the ligand and receptor could achieve low-cost adaptation during complex formation.

Further decomposition of the individual energy terms showed that the five peptides exhibited almost no covalent contribution, indicating that their recognition was essentially a typical noncovalent binding process. CTGAA showed vdW, Lipo, and Hbond values of −46.62, −8.97, and −4.51 kcal/mol, respectively. Although no single term was extremely prominent, it formed the most balanced energy combination through favorable hydrophobic contact, moderate hydrogen-bond support, and the lowest strain cost. This was consistent with its neutral small-molecule backbone composed of Cys, Thr, Gly, Ala, and Ala. The side chains of this sequence were relatively small. Ala and Gly reduced steric conflict, whereas Thr and Cys provided the necessary polar interaction sites. As a result, the peptide more readily achieved compact embedding within the receptor cavity while maintaining a low conformational burden. IDQILG showed the strongest vdW and Lipo values among the five peptides, reaching −83.33 and −17.51 kcal/mol, respectively, indicating that its binding advantage mainly arose from the large hydrophobic contact surface formed by Ile, Ile, and Leu, whereas Gln and Asp provided a certain degree of polar compensation. However, its Coulomb value reached 115.27 kcal/mol, suggesting that electrostatic matching was not ideal. Its longer chain length also increased the strain cost to 31.64 kcal/mol. Therefore, its final total binding free energy was not converted into the optimal result. KDTHP showed an H-bond value of −7.21 kcal/mol, a vdW value of −66.45 kcal/mol, and a Solv GB value of −59.75 kcal/mol, indicating that local polar recognition was not weak. However, Lys, Asp, Thr, and His together increased the polarity complexity of the sequence, whereas Pro introduced strong conformational restriction, leading to a Coulomb value of 90.59 kcal/mol and a strain cost of 37.12 kcal/mol. As a result, the net binding free energy was only −14.28 kcal/mol. This suggests that the peptide more readily formed strong local contacts, but was less able to achieve low-cost stable coordination at the overall level. QRQ displayed a distinct charge-driven energy pattern. Its Coulomb, H-bond, and Packing values were −71.91, −8.00, and −3.84 kcal/mol, respectively, all indicating strong directional recognition ability. This suggests that the Arg guanidinium group and the Gln amide groups at both ends were able to form concentrated and effective polar anchoring within the receptor pocket. At the same time, its Solv GB value reached 68.12 kcal/mol, the most unfavorable among all sequences, reflecting the substantial cost required for desolvation of a highly polar short peptide. Despite this, QRQ had a short chain length and a strain cost of only 24.59 kcal/mol, which still allowed its total binding free energy to reach −39.47 kcal/mol. This indicates that the peptide was a typical example in which strong electrostatic anchoring offset unfavorable solvation effects. EITGR showed the best no-strain binding free energy. Its Hbond, Lipo, vdW, and Solv GB values were −8.30, −10.56, −68.45, and 0.01 kcal/mol, respectively, indicating strong overall matching in terms of hydrogen bonding, hydrophobic interaction, and solvation. Based on its sequence features, Glu and Arg provided negatively and positively charged recognition endpoints, respectively; Thr provided a hydroxyl hydrogen-bonding site, Ile provided a hydrophobic contact surface, and Gly enhanced backbone flexibility. Therefore, this peptide possessed a natural advantage for constructing multipoint layered recognition. However, it was precisely this requirement for multipoint cooperation that produced the highest strain cost of 43.36 kcal/mol when the peptide achieved optimal arrangement within the receptor cavity, thereby weakening the final net binding result.

Overall, the MM-GBSA results of the five umami peptides revealed two key patterns. First, stable recognition between umami peptides and T1R1/T1R3 was not determined by a single hydrogen bond or a single electrostatic interaction, but was shaped cooperatively by multiple energy terms, including vdW, Lipo, Hbond, and Coulomb. Second, sequence length, side-chain polarity distribution, and backbone flexibility jointly determined the conversion efficiency from no-strain potential to actual total binding energy. Within this framework, CTGAA represented a low-strain balanced binding mode, QRQ represented an electrostatic anchoring mode, IDQILG reflected a hydrophobically driven binding mode, KDTHP reflected a highly polar mode with a high rearrangement cost, and EITGR showed a multipoint cooperative feature with strong intrinsic affinity but high conformational cost. These differences indicate that the interaction mechanism between umami peptides and the receptor is essentially the result of coupling among ligand chemical composition, local interactions, and overall conformational adaptation.

### 3.5. Confirmation Analysis of the Effects of Representative Umami Peptides on Umami and Other Sensory Dimensions of Non-Alcoholic Beer

**Figure 3:**
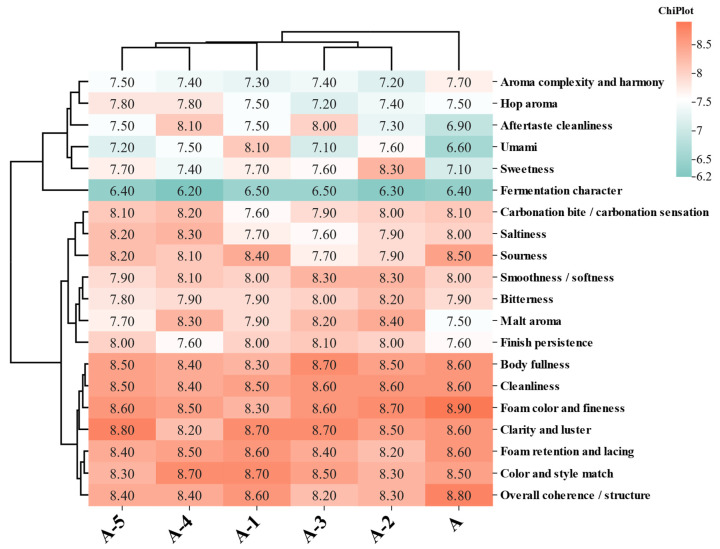
Effects of representative umami peptides on the sensory properties of non-alcoholic beer.

As shown in Figure 3, the five peptides showed clear directional consistency in their sensory effects, and all peptide-added samples differed significantly from the non-added sample (*p* < 0.05). Compared with A, all five peptide-added samples showed increases in four dimensions, namely malt aroma, sweetness, umami, and aftertaste cleanliness. The increase in umami was the most concentrated, ranging from 7.58% to 22.73%. Aftertaste cleanliness increased by 5.80% to 17.39%, sweetness by 4.23% to 16.90%, and malt aroma by 2.67% to 12.00%. In contrast, sourness, foam color and fineness, aroma complexity and harmony, and overall harmony all showed downward trends in the five samples, with decreases of 1.18% to 9.41%, 2.25% to 6.74%, 2.60% to 6.49%, and 2.27% to 6.82%, respectively. These results indicate that the main contribution of umami peptides was not the comprehensive improvement of all sensory dimensions. Instead, their effects were more concentrated on modifying the middle stage of taste, the connection of the aftertaste, and malt-related flavor, thereby shifting the originally thin and loose taste structure of non-alcoholic beer toward a more concentrated and cleaner profile. Previous studies have pointed out that non-alcoholic or low-alcohol beer often shows a more pronounced sweet taste and an overall flavor profile different from that of conventional beer. Ethanol itself is closely related to sweetness, body fullness, and aftertaste perception. Therefore, after the loss of ethanol support, the beer body often exhibits insufficient flavor support and weak mouthfeel.

In terms of the performance of individual peptides, CTGAA showed the strongest enhancement of umami. In A-1, umami increased from 6.60 to 8.10, corresponding to an increase of 22.73%. Sweetness and aftertaste cleanliness also increased by 8.45% and 8.70%, respectively. These results indicate that this short-chain small peptide was more likely to strengthen the main umami channel and the closure of the late stage. However, its improvement in body fullness and carbonation bite was limited, with decreases of 3.49% and 6.17%, respectively, indicating that its main effect was clarification of taste rather than thickening of texture. IDQILG showed a more prominent comprehensive gain. In A-2, sweetness, malt aroma, and umami increased by 16.90%, 12.00%, and 15.15%, respectively, while sourness decreased by 7.06%. This indicates that the peptide was able to enhance both malt-related flavor and a rounded sweet-umami sensation. However, overall harmony decreased by 5.68%, suggesting that its strengthening effect on local flavor exceeded its repair effect on global balance. KDTHP was more characterized by aftertaste modification. In A-3, aftertaste cleanliness increased by 15.94%, finish persistence increased by 6.58%, and this was the only treatment among the five samples that increased body fullness, with an increase of 1.16%. These results indicate that it made a certain positive contribution to late-stage extension and mouthfeel fullness. However, sourness decreased by 9.41% and overall harmony decreased by 6.82%, indicating that its modifying effect was concentrated rather than comprehensive. QRQ showed the best performance in aftertaste cleanliness. In A-4, this attribute increased from 6.90 to 8.10, corresponding to an increase of 17.39%. Umami increased by 13.64%, malt aroma by 10.67%, and hop aroma by 4.00%, indicating that this peptide was able to improve both front-stage recognition and late-stage closure and was the sequence that most clearly organized the flavor line among the five peptides. The improvement caused by EITGR was relatively moderate but more evenly distributed. Umami, sweetness, finish persistence, and hop aroma increased by 9.09%, 8.45%, 5.26%, and 4.00%, respectively, indicating that it was closer to a broad-spectrum modifier rather than a strong stimulator in a single dimension.

Combined with the sequence characteristics of the five peptides, it can be further seen that the regulation of the sensory properties of non-alcoholic lager by umami peptides showed clear structure dependence. CTGAA has a short sequence and low steric hindrance and contains the hydroxyl group of Thr and the sulfur-containing side chain of Cys. It can therefore more readily amplify the umami signal without significantly disturbing the structure of the base beer. IDQILG contains both polar sites such as Asp and Gln and hydrophobic residues such as Ile and Leu, which is more favorable for generating a composite effect in which sweet-umami sensation and malt character increase simultaneously. KDTHP contains Lys, Asp, His, and Pro, and thus combines polarity with conformational restriction. As a result, its effects are more likely to be concentrated on the adjustment of late-stage retention and oral convergence. QRQ is centered on Gln and Arg, and the combination of amide and guanidinium groups gives it strong polar recognition potential. Therefore, it showed the strongest performance in umami clarity and aftertaste cleanliness. EITGR contains the acidic residue Glu, the hydrophobic residue Ile, the polar residue Thr, the flexible residue Gly, and the basic residue Arg. Its structure is therefore more balanced, and it showed slight but synchronous improvements in multiple attributes.

Overall, all five representative umami peptides improved, to varying extents, umami expression, sweet-umami coordination, and aftertaste cleanliness in non-alcoholic lager beer. However, their effects on foam, overall harmony, and part of the mouthfeel framework were insufficient. This indicates that umami peptides are more suitable as fine-tuning factors for taste and aftertaste than as the sole means of solving all sensory defects of non-alcoholic beer.

## 4. Discussion

The present study should be understood in the broader context of flavor reconstruction in NAB. The sensory weakness of NAB is not caused by 1 missing compound. It is a combined result of low ethanol, altered volatile release, weak body support, and insufficient aftertaste continuity. Previous studies on commercial nonalcoholic lagers have shown that consumer liking is closely related to aroma quality, sweetness balance, bitterness control, mouthfeel, and overall similarity to regular beer. This means that improving NAB cannot rely only on aroma recovery or bitterness adjustment. The taste matrix also needs to be rebuilt. Peptides provide a useful entry point for this purpose. They are nonvolatile molecules, but they may affect umami, mouthfulness, taste persistence, and flavor harmony. This role is different from that of esters, higher alcohols, aldehydes, and hop-derived volatiles. It is quieter, but it may be more important for the bottom structure of taste.

The results of this study support this idea. Peptides identified in the NAB matrix were mainly short to medium oligopeptides. This length range is favorable for receptor access, water compatibility, and flexible interaction with the T1R1 and T1R3 umami receptors. The selected peptides did not share 1 simple sequence pattern. Some contained acidic residues that are often associated with umami recognition. Others showed strong predicted binding through different combinations of hydrogen bonding, electrostatic matching, and local hydrophobic contact. This suggests that peptide-mediated umami contribution in NAB is not governed by a single residue rule. It is more likely shaped by chain length, charge distribution, side chain orientation, and matrix exposure. The sensory results also need to be interpreted through this lens. The peptides did not merely increase umami intensity. They also changed aftertaste, cleanliness and overall taste balance. Such effects are consistent with studies on kokumi and taste-active peptides, which show that some peptides mainly enhance mouthfulness, continuity, and long-lasting taste rather than producing a strong independent taste by themselves.

From an R&D perspective, the practical value of these findings lies in the shift from additive thinking to matrix design. Direct addition of a purified peptide can confirm sensory potential, but industrial brewing requires a more controllable route. In real production, peptide profiles may be regulated through malt selection, mashing temperature, protease activity, yeast metabolism, fermentation control, and downstream dealcoholization or blending processes. The macromolecular composition of NAB has been linked to palate fullness and mouthfeel, which indicates that peptide regulation should be considered together with proteins, polysaccharides, dextrins, minerals, bitterness, carbonation, and volatile aroma compounds. A peptide that improves umami in a model system may not always improve beer quality in production. It may also influence haze, foam, filtration, bitterness, or stability. Therefore, peptide-based flavor reconstruction should not be treated as a single target intervention. It should be integrated with aroma retention, body construction, bitterness balancing, and sensory validation.

The computational workflow used in this study also has a practical meaning, but it should not be overstated. Machine learning prediction, molecular docking, and MM GBSA analysis can reduce the screening space and help explain possible receptor interactions. They cannot replace sensory evidence. This is especially important for NAB, where the final drinking experience is shaped by cross-modal integration among aroma, taste, mouthfeel, and aftertaste. In this study, computational screening was therefore used as a prioritization tool, while sensory validation in the NAB matrix was used to test whether the predicted peptides had real perceptual relevance. This multi-step strategy gives a more reliable basis for R&D than using sequence prediction or docking score alone.

Taken together, this study suggests that umami peptides may serve as molecular contributors to the taste base of NAB. Their value is not limited to stronger umami. A more meaningful contribution may lie in rebuilding the weak middle and late palate of NAB. This is the part that consumers often perceive as thin, short, or incomplete. Future work should move from identification to process regulation. Recombination tests, omission tests, concentration response curves, pilot-scale brewing trials, and storage stability studies are needed. These studies would clarify whether the candidate peptides can be generated or retained through brewing control rather than added externally. Such work would make peptide-guided flavor reconstruction more practical for NAB product development.

## 5. Limitations

### 5.1. Limitations of Molecular Docking in Sensory Prediction

Molecular docking provided useful structural clues in this study. It helped locate possible binding poses between the candidate peptides and the T1R1 and T1R3 umami receptor model. It also allowed a preliminary comparison of hydrogen bonds, hydrophobic contacts, electrostatic interactions, and binding energy. Even so, docking should not be read as direct proof of taste activity. A favorable docking score only suggests that a peptide may fit into a receptor binding region. It does not mean that the peptide will certainly produce umami perception in a real beer matrix.

This limitation is especially important for peptide taste research. Sensory perception is not controlled by receptor binding alone. It is shaped by peptide concentration, solubility, bitterness, sweetness, acidity, saltiness, ethanol background, carbonation, and interactions with other beer components. A peptide with strong predicted receptor affinity may be present at a level below its sensory threshold. Another peptide may bind weakly in silico but still contribute to taste through matrix interaction or synergistic effects. In beer, this gap can become larger. The matrix is chemically crowded. Organic acids, amino acids, sugars, nucleotides, minerals, bitter substances, and aroma compounds may all change the final taste response.

Docking also simplifies receptor behavior. The T1R1 and T1R3 receptors are a flexible protein system. Its active conformation may change after ligand binding. A static docking model cannot fully describe this movement. Water molecules, membrane environment, receptor activation, downstream signaling, and oral physiological conditions are also difficult to represent completely. Scoring functions have another weakness. They are useful for ranking candidates, but they do not always give accurate binding free energy. This is a known limitation of docking-based screening, because receptor flexibility, ligand sampling, solvation, and entropy are often simplified during calculation.

For these reasons, docking was used here as a screening and interpretation tool, not as the final criterion for umami activity. The predicted binding mode was combined with machine learning prediction, peptide identification, and sensory validation. This combined strategy reduced the risk of overinterpreting computational results. It also made the conclusion closer to real taste perception in non-alcoholic beer. Still, the present study has room for improvement. Future work should include molecular dynamics simulation, receptor activation assays, concentration response tests, and sensory recombination experiments. These approaches would help clarify whether the predicted receptor interaction can be translated into a stable and perceivable umami contribution.

### 5.2. Limitations Related to Peptide Structure Activity Relationships and Brewing Application

The present study identified several potential umami peptides in NAB. It also linked peptide sequences with computational prediction and sensory verification. Yet the structure–activity relationship of beer peptides remains only partly explained. Umami activity cannot be judged from 1 residue or 1 simple motif. Acidic residues may support umami perception. Polar residues may improve water solubility. Hydrophobic residues may strengthen receptor contact, but they may also bring bitterness. Terminal residues also matter. A small change at the N terminal or C terminal may alter receptor recognition, taste intensity, and aftertaste quality. These effects are not linear. They depend on sequence length, charge distribution, spatial flexibility, and matrix behavior.

This point is important for interpreting the peptides screened in this study. The selected peptides showed potential umami contribution in the NAB system. They should not be viewed as universal umami markers. A peptide that works in NAB may behave differently in soy sauce, cheese, meat broth, or ordinary alcoholic beer. The same sequence may also show different sensory effects at different concentrations. Below its taste threshold, it may have little direct impact. At a higher level, it may change thickness, continuity, bitterness, or overall balance. For this reason, the identified peptides should be understood as matrix-related contributors rather than isolated taste units.

Computational prediction also has clear boundaries. Machine learning models are useful for narrowing a large peptide pool. They can capture residue composition, sequence pattern, and known taste-related features. Still, their judgment depends on the training data. Current umami peptide datasets are still limited in size and food origin. They also contain uneven negative samples. Many models give a binary output or a probability score. This output does not equal sensory intensity. It cannot provide taste threshold, temporal persistence, mouthfeel effect, or interaction with beer components. A high prediction score therefore means priority for validation. It does not mean a confirmed umami function. This is consistent with current peptide taste prediction studies, which treat sequence-based models as screening tools rather than replacements for sensory testing.

The practical meaning of brewing should also be treated carefully. These results suggest that peptide regulation may become a useful route for improving the weak body and limited taste continuity of NAB. Yet direct peptide addition is not the only possible strategy. It may not even be the best industrial route. Brewers may instead regulate endogenous peptide formation through malt selection, mashing conditions, protease activity, yeast metabolism, fermentation control, and dealcoholization management. Such process-based regulation is closer to real production. It may also reduce cost and improve label acceptance.

There are tradeoffs. Higher peptide abundance may improve umami, roundness, or mouthfeel. It may also affect foam, haze, filtration efficiency, colloidal stability, and flavor cleanliness. Some peptides may contribute bitterness or a lingering aftertaste. Others may bind with polyphenols, proteins, or minerals and change beer stability. The brewing value of an umami peptide therefore depends on more than taste enhancement. It must be evaluated together with process feasibility, batch stability, sensory balance, and product style.

Future work should move from peptide discovery to controlled structure–activity verification. Synthetic analogues can be designed by replacing key residues, changing terminal amino acids, or adjusting peptide length. Their thresholds and intensity curves should be measured in NAB. Recombination and omission tests are also needed. Pilot-scale brewing trials would further show whether these peptides can be regulated through process design. This step is essential. It would turn computationally screened peptides into practical flavor factors for NAB development.

### 5.3. Limitations in Interpreting Cross-Attribute Effects of Individual Peptides

The sensory effects of individual peptides were interpreted cautiously in this study. Although the addition of selected peptides changed several sensory scores, these changes should not be understood as direct evidence that a single peptide independently produced sweetness or aroma. Peptides are mainly nonvolatile taste-active compounds. Their direct contribution is more closely related to umami, mouthfulness, continuity, and aftertaste. In contrast, sweetness-related or aroma-related changes are more likely to reflect perceptual modulation within the NAB matrix. Umami substances can modulate sweet taste, enhance salty taste, and suppress bitterness, but these effects are usually matrix-dependent and concentration-dependent [55]. Kokumi peptides have also been reported to enhance mouthfulness and long-lasting taste rather than acting as simple single taste compounds [56]. Therefore, in the present study, changes in sweetness and aroma scores were regarded as shifts in perceived flavor balance and aroma impression. They were not taken as proof of direct activation of sweet taste receptors or olfactory pathways by the peptides. This interpretation is also consistent with the concept of taste aroma interaction, in which taste, aroma, and oral texture are integrated during eating or drinking and jointly shape the overall flavor impression [57,58]. Further recombination, omission, concentration response, and volatile release experiments are still needed to clarify whether these cross-attribute changes arise from direct taste modulation, indirect matrix effects, or panel-based multisensory integration.

## 6. Conclusions

This study focused on key problems in non-alcoholic beer, including insufficient body support, weak umami expression, and incomplete aftertaste layering. A novel screening pathway for umami peptides was established by integrating machine learning prediction, receptor-recognition analysis, and sensory validation. The potential role of umami peptides in flavor reconstruction of non-alcoholic beer was also revealed in a relatively systematic manner from a complex brewing matrix. At the peptide-identification level, a total of 2081 unique sequences were obtained, indicating the presence of a considerable and compositionally complex peptide library in non-alcoholic beer. Most of these were short peptides of 3 to 5 aa and simultaneously showed structural features of high P, high Q, and coexisting hydrophobic residues. Through dual-model screening based on Umami-Transformer and iUmami-SCM, 122 potential umami peptides were identified, reflecting that a group of short-chain oligopeptides with potential umami contribution does indeed exist in non-alcoholic beer.

At the receptor-recognition level, the extracellular recognition-domain model of T1R1/T1R3 constructed with 1EWK as the template showed good stereochemical rationality and docking applicability. The Ramachandran, ERRAT, and VERIFY results all indicated that the model could support subsequent virtual screening. The docking results showed that 117 peptides could form stable predicted binding with the receptor, and that the successfully docked peptides were mainly tetrapeptides and pentapeptides, suggesting that medium-short peptides better matched the spatial accommodation characteristics of the receptor pocket. The five peptides further screened, namely CTGAA, IDQILG, KDTHP, QRQ, and EITGR, not only ranked highly in docking score but also showed good structural representativeness in peptide length, polar-residue composition, and hydrophobic backbone. Their recognition by T1R1/T1R3 did not rely on a single site. Instead, hydrogen-bonding and electrostatic anchoring networks were constructed within the Venus flytrap domain through residues such as Ser, Gln, Asn, Gly, Thr, His, Glu, and Asp, whereas hydrophobic or aromatic residues such as Ile, Leu, Val, Tyr, and Trp provided local burial and conformational buffering. In this way, a cooperative recognition mode was formed, dominated by hydrogen bonding and electrostatic interactions and assisted by hydrophobic support. This result is consistent with previous receptor studies of umami peptides, which have shown that T1R1/T1R3 mainly relies on hydrogen-bonding, electrostatic, and hydrophobic interactions to recognize ligands.

The MM-GBSA results further indicated that stable binding between umami peptides and the receptor depended not only on strong local interactions but also on the efficiency of overall conformational adaptation. CTGAA showed the lowest total binding free energy, at −45.21 kcal/mol, and exhibited a low-strain and relatively balanced binding feature. EITGR showed the lowest no-strain binding free energy, at −72.14 kcal/mol, indicating strong intrinsic affinity potential, but its higher conformational adjustment cost weakened the final net binding advantage. These results indicate that the essence of umami peptide receptor recognition is the combined coupling of local energy terms and overall adaptation cost.

At the sensory-validation level, all five representative umami peptides improved the umami expression, sweet-umami coordination, and aftertaste cleanliness of non-alcoholic beer after individual addition. Among these effects, umami increased by 7.58% to 22.73%, aftertaste cleanliness by 5.80% to 17.39%, sweetness by 4.23% to 16.90%, and malt aroma by 2.67% to 12.00%. CTGAA showed the most obvious enhancement of umami. QRQ showed the most prominent improvement in aftertaste cleanliness. IDQILG showed good overall performance in sweet-umami sensation and malt support. KDTHP was more oriented toward late-stage extension and body-fullness modification. EITGR showed relatively balanced improvements in multiple attributes.

Therefore, CTGAA, IDQILG, KDTHP, QRQ, and EITGR can be identified as representative novel umami peptides in the non-alcoholic beer system investigated in this study. They can not only directly participate in umami enhancement, but can also positively contribute to the reconstruction of the sensory framework of non-alcoholic beer by regulating sweet-umami balance, malt support, and aftertaste integration. Looking forward, further studies are still needed on individual peptide threshold determination, peptide-peptide synergistic effects, and process-enrichment validation in real brewing systems. These efforts should be combined with receptor-activation evidence and larger consumer preference datasets to promote the transition of such novel umami peptides from mechanistic discovery to practical application in non-alcoholic beer flavor optimization.

## Figures and Tables

**Table 1:** Qualitative identification of peptides in non-alcoholic beer and their potential contribution to umami flavor.

Number	Peptide	−10LgP	Mass	Length	*m*/*z*	RT	Area	PTM	ALC (%)	UmamiPredict	Prediction (Umami Score)
1	AAAIA	22.96	415.24	5	416.25	55.15	5250.00			Umami	0.97
2	AAAVP	21.29	427.24	5	429.19	16.72	324,000.00			Umami	0.98
3	AACH	16.70	400.15	4	401.16	49.10	1030.00			Umami	0.96
4	AACT	18.01	406.15	4	407.20	4.75	2310.00	Acetylation (Protein N-term)		Umami	0.93
5	AADF	27.36	422.18	4	423.19	12.40	6610.00			Umami	0.97
6	AAGGAY	20.78	508.23	6	509.23	8.17	15,900.00			Umami	0.71
7	AAIAA	17.52	415.24	5	416.25	55.10	5250.00			Umami	0.97
8	AAKKK	16.45	544.37	5	545.38	49.79	13,000.00			Umami	0.80
9	AATAR	15.57	489.25	5	490.69	13.40	9550.00	Deamidation (R)		Umami	0.93
10	AATM	21.67	392.17	4	393.18	42.16	314.00			Umami	0.86
11	AATP	15.60	358.19	4	359.32	45.36	337.00			Umami	0.86
12	ACHA	15.54	400.15	4	401.29	5.88	9100.00			Umami	0.75
13	ADASV	18.07	461.21	5	462.21	43.39	453.00			Umami	0.73
14	AEIIIP	15.52	654.40	6	655.40	43.50	4130.00			Umami	0.77
15	AEILLP	19.58	654.40	6	655.40	43.49	4130.00			Umami	0.75
16	AGAAT	16.75	389.19	5	390.20	43.98	5590.00			Umami	0.70
17	AHAR	15.51	454.23	4	455.23	1.89	20,600.00	Deamidation (R)		Umami	0.83
18	ARAAC	16.05	490.23	5	491.33	44.36	371.00			Umami	0.97
19	ARGGK	20.17	488.27	5	489.34	56.39	93.20	Deamidation (R)		Umami	0.85
20	ARIP	25.58	455.29	4	456.73	9.83	3470.00			Umami	0.85
21	ARIQL	15.84	600.36	5	601.36	9.86	258.00	Deamidation (R)		Umami	0.83
22	ARNK	18.06	488.27	4	489.28	56.42	93.20	Deamidation (R)		Umami	0.86
23	ARPC	19.75	446.19	4	447.17	58.65	1740.00	Deamidation (R)		Umami	0.86
24	ASCH	21.51	416.15	4	417.15	41.73	238.00			Umami	0.77
25	ASI	28.73	331.17	3	332.18	10.92	17,200.00	Acetylation (Protein N-term)		Umami	0.74
26	ASTE	15.32	406.17	4	407.27	58.41	92,900.00			Umami	0.73
27	AVSH	15.58	412.21	4	413.21	1.68	69,000.00			Umami	0.84
28	AVSI	26.89	388.23	4	389.24	11.41	18,000.00			Umami	0.84
29	AVSMPP	17.60	616.29	6	617.79	39.71	515.00	Oxidation (M)		Umami	0.70
30	CADHV		585.22	5	586.23	2.76	1507.25	Acetylation (Protein N-term)	92.4	Umami	0.79
31	CTGAA	16.31	421.16	5	422.17	56.49	1730.00			Umami	0.88
32	CTRK	15.25	506.26	4	507.27	58.32	1020.00			Umami	0.70
33	DAAP	28.69	372.16	4	373.17	3.53	17,000.00			Umami	0.83
34	DC	18.85	277.05	2	278.06	25.36	739.00	Acetylation (Protein N-term); Half of a disulfide bridge		Umami	0.79
35	DIAR	25.26	474.24	4	475.29	42.25	7990.00	Deamidation (R)		Umami	0.86
36	DIVSAR	19.46	659.36	6	660.37	10.36	5360.00			Umami	0.93
37	DKDP	22.52	473.21	4	474.22	4.65	12,900.00			Umami	0.81
38	DP	17.01	272.10	2	273.11	56.07	1630.00	Acetylation (Protein N-term)		Umami	0.75
39	DQIIP	22.77	584.32	5	585.32	33.48	12,200.00			Umami	0.82
40	DQPRT	18.60	616.28	5	617.29	29.10	1250.00	Deamidation (R)		Umami	0.77
41	DSTYQP	17.95	709.29	6	710.30	12.24	5330.00			Umami	0.74
42	DVADAAGT	18.44	718.31	8	720.87	38.33	260.00			Umami	0.72
43	DVSP	26.68	416.19	4	417.20	8.58	13,000.00			Umami	0.86
44	EAAD	17.36	386.14	4	387.30	55.10	91.50	Pyro-glu from E		Umami	0.97
45	EAAHP	17.52	523.24	5	524.30	41.51	392.00			Umami	0.75
46	EAATQ	17.05	518.23	5	519.14	2.96	1470.00			Umami	0.78
47	EAAYPS	16.05	636.28	6	637.28	39.42	1230.00			Umami	0.96
48	EI	35.56	260.14	2	261.14	11.36	69,500.00			Umami	0.93
49	EIA	23.11	313.16	3	314.17	57.24	4640.00	Pyro-glu from E		Umami	0.76
50	EII	25.46	355.21	3	356.22	40.37	8980.00	Pyro-glu from E		Umami	0.76
51	EIKYQ	17.94	679.35	5	680.36	46.29	63.50			Umami	0.71
52	EIP	26.75	339.18	3	340.19	25.53	29,600.00	Pyro-glu from E		Umami	0.85
53	EITD	15.46	476.21	4	478.29	55.55	1180.00			Umami	0.79
54	EITGR	25.41	556.30	5	557.30	12.09	12,600.00	Pyro-glu from E		Umami	0.71
55	EKDGA	16.65	518.23	5	519.24	2.68	1470.00			Umami	0.72
56	ESPAA	18.33	455.20	5	456.21	3.99	484,000.00	Pyro-glu from E		Umami	0.79
57	ESYP	32.94	476.19	4	477.20	16.24	10,500.00	Pyro-glu from E		Umami	0.89
58	EVSH	16.53	470.21	4	471.22	43.68	1270.00			Umami	0.84
59	EYST	15.21	498.20	4	499.20	4.82	10,300.00			Umami	0.86
60	GAAVE	24.03	445.22	5	446.22	4.46	14,400.00			Umami	0.88
61	GSK	27.42	290.16	3	291.17	54.38	1150.00			Umami	0.71
62	GTAA	17.04	318.15	4	319.16	47.24	758.00			Umami	0.89
63	GVAA	26.87	316.17	4	317.21	58.22	309.00			Umami	0.74
64	HPST	19.97	440.20	4	441.21	42.66	2850.00			Umami	0.80
65	HVAA	24.41	396.21	4	397.22	47.90	1640.00			Umami	0.86
66	IAEIP	21.02	541.31	5	542.35	28.64	2400.00			Umami	0.83
67	IDMIP	18.79	587.30	5	588.31	41.18	11,400.00			Umami	0.73
68	IDQILG	15.22	657.37	6	658.38	39.68	893.00			Umami	0.71
69	IDQP	23.34	471.23	4	472.24	8.33	22,800.00			Umami	0.85
70	IGAR	27.85	415.25	4	416.26	5.25	6430.00			Umami	0.81
71	IHAD	24.02	454.22	4	455.23	45.53	1670.00			Umami	0.79
72	INNAA	26.97	501.25	5	502.26	4.94	17,600.00			Umami	0.82
73	ISPSK	26.99	530.31	5	531.31	4.99	2460.00			Umami	0.79
74	KDTHP	18.10	596.29	5	597.30	4.70	3670.00			Umami	0.75
75	KKDI	28.63	502.31	4	503.32	51.17	307.00			Umami	0.77
76	KSVS	19.78	419.24	4	420.25	41.76	579.00			Umami	0.84
77	KVSS	26.53	419.24	4	420.20	41.76	579.00			Umami	0.88
78	LGAAVP	15.16	526.31	6	527.37	49.78	407.00			Umami	0.82
79	MID	33.49	377.16	3	378.17	9.56	3630.00			Umami	0.85
80	MIEIP	25.51	601.31	5	602.32	41.20	4600.00			Umami	0.85
81	MKD	28.73	408.17	3	409.24	9.19	3550.00	Oxidation (M)		Umami	0.95
82	MPSE	25.17	462.18	4	463.19	46.30	2670.00			Umami	0.80
83	NAAAP	19.92	442.22	5	444.28	8.41	3390.00			Umami	0.93
84	NIN	28.81	359.18	3	360.19	4.20	18,500.00			Umami	0.78
85	NINI	17.15	474.23	4	475.18	32.32	102.00	Deamidation (NQ);Deamidation (NQ)		Umami	0.79
86	NIT	17.93	347.17	3	348.66	5.18	40,400.00	Deamidation (NQ)		Umami	0.82
87	PARSI	17.43	542.32	5	543.32	4.65	1550.00			Umami	0.78
88	PE	17.42	286.12	2	287.13	45.06	212.00	Acetylation (Protein N-term)		Umami	0.94
89	PEHA	20.93	452.20	4	453.21	4.59	1300.00			Umami	0.82
90	PGDPAR	16.87	611.30	6	612.78	21.90	112.00			Umami	0.77
91	PPEIP	18.88	551.30	5	552.29	9.46	4950.00			Umami	0.82
92	PSCT	22.53	406.15	4	407.20	4.81	2310.00			Umami	0.80
93	QACA	18.81	374.13	4	375.13	46.57	1310.00	Pyro-glu from Q		Umami	0.95
94	QAELIIP	25.55	765.43	7	766.43	47.73	1000.00	Pyro-glu from Q		Umami	0.80
95	QPAAP	16.46	482.25	5	483.23	40.10	253.00			Umami	0.75
96	QPQPEPS	18.21	764.33	7	765.35	9.32	2230.00	Pyro-glu from Q		Umami	0.74
97	QQAE	17.41	516.22	4	518.28	46.03	28.70	Acetylation (Protein N-term)		Umami	0.94
98	QQAELIIP	33.36	893.49	8	447.75	46.20	2740.00	Pyro-glu from Q		Umami	0.76
99	QRQ	15.72	413.20	3	414.16	3.37	498.00	Pyro-glu from Q		Umami	0.75
100	QSPAA	16.25	455.20	5	456.21	3.54	484,000.00	Pyro-glu from Q		Umami	0.79
101	QVSTE	16.05	562.26	5	563.75	30.05	905.00			Umami	0.81
102	RKHE	15.52	568.31	4	285.16	13.90	3960.00			Umami	0.70
103	SGICAAD	19.18	635.26	7	636.27	2.61	11,200.00			Umami	0.76
104	SVSD	24.10	406.17	4	407.18	58.01	92,900.00			Umami	0.84
105	TCQA	17.62	421.16	4	422.17	56.54	1730.00			Umami	0.82
106	TFD	17.95	381.15	3	382.16	54.27	895.00			Umami	0.86
107	TTCAA	21.42	464.18	5	465.19	44.26	544.00	Half of a disulfide bridge		Umami	0.95
108	TTHHP	15.02	591.28	5	592.28	9.53	4280.00			Umami	0.72
109	TVDPST	17.60	618.29	6	619.81	21.01	31.60			Umami	0.73
110	TVSP	28.82	402.21	4	403.22	8.54	105,000.00			Umami	0.73
111	TYTV	15.72	482.24	4	484.32	13.31	24,400.00			Umami	0.86
112	VDV	25.49	331.17	3	331.69	5.30	7470.00			Umami	0.71
113	VPSQP	28.11	526.28	5	527.28	9.43	21,700.00			Umami	0.73
114	VSAAH	21.86	483.24	5	484.27	10.63	2440.00			Umami	0.96
115	VSIE	20.89	446.24	4	447.72	10.81	3480.00			Umami	0.84
116	VSIIP	22.33	527.33	5	528.34	39.26	918.00			Umami	0.81
117	VSIVI	20.34	529.35	5	530.35	41.09	1520.00			Umami	0.76
118	VSTEP	24.97	531.25	5	532.26	8.96	24,500.00			Umami	0.81
119	VTPSK	25.85	530.31	5	531.31	5.29	2460.00			Umami	0.86
120	YPEQP	24.00	632.28	5	633.29	12.38	20,000.00			Umami	0.86
121	YPESQQP	28.49	847.37	7	848.38	10.57	7590.00			Umami	0.75
122	YPVD	20.35	492.22	4	493.23	11.53	7740.00			Umami	0.82

Note: −10LgP indicates the confidence score for peptide identification. Mass indicates the molecular mass of the peptide. Length indicates the peptide length. *m*/*z* indicates the mass-to-charge ratio. RT indicates the retention time. Area indicates the peak area. PTM indicates post-translational modification. ALC (%) indicates average local confidence. UmamiPredict indicates the predicted class of umami peptides. Prediction (umami score) indicates the predicted umami score. The renderings of ALC and related PEAKS terminology follow the PEAKS documentation, and *m*/*z* follows the standard mass spectrometry convention.

**Table 3:** MM-GBSA binding energies of five umami peptides with their receptor.

Peptide	MMGBSA dG Bind	MMGBSA dG Bind (NS)	MMGBSA dG Bind (NS) Coulomb	MMGBSA dG Bind (NS) Covalent	MMGBSA dG Bind (NS) Hbond	MMGBSA dG Bind (NS) Lipo	MMGBSA dG Bind (NS) Packing	MMGBSA dG Bind (NS) Solv GB	MMGBSA dG Bind (NS) vdW
CTGAA	−45.21	−65.74	1.62	0.00	−4.51	−8.97	0.00	−7.27	−46.62
IDQILG	−28.83	−60.47	115.27	0.00	−5.37	−17.51	−3.01	−66.51	−83.33
KDTHP	−14.28	−51.40	90.59	0.00	−7.21	−7.48	−1.11	−59.75	−66.45
QRQ	−39.47	−64.06	−71.91	0.00	−8.00	−5.64	−3.84	68.12	−42.78
EITGR	−28.78	−72.14	16.50	0.00	−8.30	−10.56	−1.34	0.01	−68.45

Note: All energy values are expressed in kcal/mol. MMGBSA dG Bind denotes the total binding free energy calculated by Prime MM-GBSA. MMGBSA dG Bind (NS) denotes the no-strain binding free energy excluding the receptor and ligand strain terms. MMGBSA dG Bind (NS) Coulomb, Covalent, Hbond, Lipo, Packing, Solv GB, and vdW represent the Coulomb energy, covalent binding energy, hydrogen-bond correction term, lipophilic interaction energy, π–π stacking correction term, Generalized Born electrostatic solvation energy, and van der Waals energy contributions to the no-strain binding free energy, respectively. The renderings of MMGBSA dG Bind, MMGBSA dG Bind (NS), Solv GB, and vdW are consistent with Prime MM-GBSA terminology.

## Data Availability

The original contributions presented in this study are included in the article/Supplementary Materials. Further inquiries can be directed to the corresponding author.

## Supplementary Materials

The following supporting information can be downloaded at: https://www.mdpi.com/article/10.3390/foods15101671/s1, Supplementary S1. Polypeptide FASTA Format Converter; Supplementary S2. Peptide Basic Chemical Symbol Calculator; Supplementary S3. Umami peptide prediction tool based on the iUmami-SCM model; Supplementary S4. Objective scoring tool for beer sensory evaluation; Supplementary S5. Online beer review corpus and multidimensional scoring of beer sensory attributes; Supplementary S6. Informed consent form; Supplementary S7. Detailed description of the scoring criteria for different sensory evaluation dimensions of non-alcoholic beer.
