# Supplementary material for: Mining and Validation of Novel Umami Peptides in Non-Alcoholic Beer by Integrating Machine Learning Prediction, Molecular Docking, and Sensory Validation, and Their Multidimensional Sensory Impacts on Beer Body
Source: Foods. 2026 May 11;15(10):1671. doi: 10.3390/foods15101671 (PMC13205247; doi:10.3390/foods15101671)
Supplement: Supplementary file 1 [file foods-15-01671-s001.zip › Supplementary S2 Peptide Basic Chemical Symbol Calculator.html]

Peptide Basic Chemical Symbol Calculator


# Peptide Basic Chemical Symbol Calculator

Designed by: Wu Yashuai; Supervised by: Zhao Dongrui.

Input one or more amino-acid sequences (single-letter codes). Each non-empty line or token becomes a separate row in the results.

## Input

Amino acid sequence(s) (supports whitespace, comma, semicolon, or newline separation; ignores FASTA headers that start with >)


Convert
Clear
Download CSV

Self-check: pending…

Notes: This generates a practical peptide SMILES for canonical, unmodified L-amino acid sequences with free N- and C-termini (no acetylation/amidation, no PTMs).
It does not model disulfide bonds or cyclization.

## Converted output

Output box (sequence → SMILES and calculated properties)


Tip: After conversion, use Download CSV to export all rows with sequential numbering.

## Results table

| No. | Sequence | SMILES | Mol. Weight | Hydrophobicity | Isoelectric Point |
| --- | --- | --- | --- | --- | --- |
| No results yet. | | | | | |

Supported amino acids (single-letter): A, C, D, E, F, G, H, I, K, L, M, N, P, Q, R, S, T, V, W, Y.
Invalid characters will be flagged per row so that exported CSV row counts always match the input row counts.
