# Supplementary material for: Mining and Validation of Novel Umami Peptides in Non-Alcoholic Beer by Integrating Machine Learning Prediction, Molecular Docking, and Sensory Validation, and Their Multidimensional Sensory Impacts on Beer Body
Source: Foods. 2026 May 11;15(10):1671. doi: 10.3390/foods15101671 (PMC13205247; doi:10.3390/foods15101671)
Supplement: Supplementary file 1 [file foods-15-01671-s001.zip › Supplementary S3 Umami peptide prediction tool based on the iUmami-SCM model.html]

Umami Peptide Predictor


# Umami Peptide Predictor

Enter amino acid sequences below, one per line (maximum 5000 lines). The sequences should be provided in SMILES or one‑letter amino acid format. Only the twenty standard amino acid characters (`ACDEFGHIKLMNPQRSTVWY`) will contribute to the prediction; other characters will be ignored.

Predict
