# Supplementary material for: Mining and Validation of Novel Umami Peptides in Non-Alcoholic Beer by Integrating Machine Learning Prediction, Molecular Docking, and Sensory Validation, and Their Multidimensional Sensory Impacts on Beer Body
Source: Foods. 2026 May 11;15(10):1671. doi: 10.3390/foods15101671 (PMC13205247; doi:10.3390/foods15101671)
Supplement: Supplementary file 1 [file foods-15-01671-s001.zip › Supplementary S4 Objective scoring tool for beer sensory evaluation.html]

Beer Sensory Evaluation Tool


# Beer Sensory Evaluation Tool

Beer Sensory Objectification
Beer Sensory Scoring Description

## Beer Sensory Objectification

Please evaluate the beer you are drinking with reference to the following 20 dimensions. Of course, if you feel that some sensory dimensions are not present, you may skip them. The most important thing is to write down your true feelings. Reference dimensions: Color & Style Matching, Clarity & Brilliance, Foam Color & Fineness, Foam Retention & Lacing, Malt Aroma, Hop Aroma, Fermentation Characteristic Aroma, Aroma Layering & Harmony, Cleanliness, Sweetness, Bitterness, Acidity, Saltiness, Umami, Body Fullness, Carbonation/Prickling Sensation, Smoothness/Softness, Body Balance/Structure, Aftertaste Cleanliness, Finish Persistence.；色泽与风格匹配、清亮度与光泽、泡沫色泽与细腻度、泡持性与挂杯、麦芽香、酒花香、发酵特征香、香气层次与协调、洁净度、甜、苦、酸、咸、鲜、酒体丰满度、杀口/碳酸感、顺滑/柔和度、酒体协调/结构、回味洁净度、余韵持久性

Enter beer sensory description:

Get Sensory Score

## Beer Sensory Scoring Description

Enter scores (1–9) for each sensory dimension:

Generate Discussion


Designer: Wu Yashuai, Chen Yiyuan; Instructor: Zhao Dongrui
