# Supplementary material for: Mining and Validation of Novel Umami Peptides in Non-Alcoholic Beer by Integrating Machine Learning Prediction, Molecular Docking, and Sensory Validation, and Their Multidimensional Sensory Impacts on Beer Body
Source: Foods. 2026 May 11;15(10):1671. doi: 10.3390/foods15101671 (PMC13205247; doi:10.3390/foods15101671)
Supplement: Supplementary file 1 [file foods-15-01671-s001.zip › Supplementary S6 Informed consent form.pdf]

Informed consent form

Dear Sir/Madam,

We would like to invite you to participate in a scientific study entitled "Mining and validation of novel umami peptides in non-alcoholic beer by integrating machine learning prediction, molecular docking, and sensory validation, and their multidimensional sensory impacts on beer body"

Before deciding whether to participate in this study, please read the following information carefully. It will help you understand the content of the study, why it is being conducted, and the possible benefits, risks, and discomforts that may arise from your participation.

The study is introduced as follows:

1. Research background

Beer flavor perception depends not only on volatile aroma compounds, but also significantly on non-volatile taste-active substances. In recent years, studies have shown that some umami peptides may contribute to mouthfulness, harmony, and the overall layering of beer flavor. To further verify the sensory contribution of representative umami peptides in a real beer system, this study uses the original beer sample from the same production batch as the base and separately adds five representative umami peptides, namely CTGAA, IDQILG, KDTHP, QRQ, and EITGR, to construct a comparable sample system before and after addition. Blind sensory evaluation will then be carried out to investigate their effects on the sensory performance of the beer body. Since this type of integrated perception still cannot be fully replaced by instrumental analysis or theoretical prediction, quantitative data from trained sensory assessors are required.

2. Study participants and eligibility criteria

Number of volunteers: A total of 10 participants, all of whom have received standardized sensory evaluation training.

Required abilities: Participants should be able to understand the evaluation requirements of this study, be familiar with the basic procedures of beer sensory evaluation, and independently complete sample scoring and recording as required.

Basic requirements: No obvious olfactory or gustatory impairment, no recent severe cold, oral disease, or acute rhinitis episode, no alcohol consumption on the day of testing, and good mental condition.

Situations in which participation is not recommended or exclusion applies (please inform us proactively if any apply)

(1). Allergy to alcohol or obvious intolerance to beer raw materials or ingredients

(2). Pregnancy or lactation

(3). Medical advice restricting alcohol consumption, or recent use of medications that may affect taste, smell, or make alcohol exposure inadvisable

(4). Plans to drive, work at heights, or operate precision or hazardous machinery shortly after the test; if safety cannot be ensured, participants may choose to sip and spit or reschedule participation

3. Research objectives

(1). To obtain sensory evaluation data for the original beer sample A and the five umami peptide-added samples A-1, A-2, A-3, A-4, and A-5, and to generate comparable quantitative results.

(2). To verify the specific contribution of different representative umami peptides to beer sensory characteristics through a single-variable addition design, thereby providing a basis for subsequent mechanistic analysis and interpretation of the results.

(3). To conduct the test on the premise of full informed consent and voluntary participation; participants may withdraw at any time, and personal information will be used only for research management and will be anonymized.

4. Specific procedures and workflow

Sample preparation and coding (blind design)

o All experimental samples are taken from the same batch of product, and the original beer sample is labeled as A.

o On this basis, the five representative umami peptides CTGAA, IDQILG, KDTHP, QRQ, and EITGR are each individually added to the original beer sample, with each treatment consisting of adding 4 mg of the umami peptide standard to 200 mL of the original beer sample, thereby ensuring that only the representative umami peptide affects the sensory properties of the beer.

o The beer samples with added umami peptides are labeled A-1, A-2, A-3, A-4, and A-5, respectively. During formal evaluation, random numerical codes will be used and samples will be presented in random order to reduce the influence of subjective expectations on the results.

Evaluation content

o The evaluation will focus on differences in overall taste performance, body fullness, harmony, mouthfulness, and flavor layering.

o During evaluation, assessors should judge whether the addition of umami peptides causes changes in beer flavor perception, as well as the direction and extent of those changes, by comparing the sensory performance of the original beer sample and each treated sample.

Scoring method

o A standardized sensory evaluation form will be used for recording in this study.

o Assessors are required to make independent judgments on the sensory performance of each sample according to the training requirements and must not discuss scoring results with others.

o All samples will be evaluated under identical conditions to ensure consistency and comparability of the test.

Recommended workflow for a single test session (subject to actual arrangement)

o Preparation and instruction: approximately 5 minutes, including confirmation of testing requirements, contraindications, and evaluation precautions.

o Blind evaluation: approximately 15–25 minutes, during which assessors evaluate samples presented in random order; water will be provided between samples, with appropriate intervals, to reduce taste fatigue.

o Completion: approximately 5 minutes, including submission of the scoring sheet and checking whether all records are complete.

5. What you need to do if you participate in the study

(1). Within 1 hour before testing, avoid spicy, high-sugar, or strongly flavored foods and beverages; avoid smoking and drinking; and try not to use perfume or strongly scented skincare products.

(2). During the test, please complete the sensory evaluation independently and do not exchange opinions or give hints to others.

(3). If you experience dizziness, nausea, palpitations, oral irritation, or any other discomfort, please inform the researchers immediately. You may pause or withdraw from the test without any adverse consequences.

6. Possible benefits of participating in this study

Participation in this study may help you understand the sensory role of representative umami peptides in a beer system and directly perceive the effects of different umami peptides on beer body, harmony, and flavor layering. The anonymized data obtained will be used to support sensory validation of umami peptides, result analysis, and subsequent studies, thereby providing a basis for beer flavor regulation and related quality evaluation research. The entire study process follows the basic ethical principles of informed consent, voluntary participation, the right to withdraw at any time, and privacy protection, with reference to the World Medical Association *Declaration of Helsinki* and relevant standards for sensory evaluation.

7. Possible adverse reactions, risks, and preventive measures associated with participation

This study involves sensory evaluation of beer samples, and the overall risk is low. Since the samples contain alcohol, a small number of participants who are sensitive to alcohol may experience mild dizziness, oral irritation, or temporary discomfort. Tasting multiple samples in succession may also cause short-term taste fatigue or temporary dullness of oral sensation. To reduce risks, the basic health condition of participants

will be confirmed before the experiment, and water and other oral cleansing conditions will be provided during testing to minimize carryover effects. The testing environment will be kept safe, quiet, and controllable. If you experience obvious discomfort during the evaluation, you may immediately pause or withdraw, and the researchers will take appropriate measures when necessary. All research data will be recorded and stored anonymously, and personal privacy will be protected.

8. Explanation of costs

You do not need to pay any fees to participate in this study. All samples used for sensory testing and related recording materials will be provided by the research team.

9. Compensation for participation, including injury compensation

You will receive appropriate compensation in appreciation of your participation, and the specific standard will be subject to the study arrangement. All samples used in the study will be uniformly prepared and managed according to the experimental requirements. If physical discomfort or any other injury occurs as a result of participating in this study, the research team will provide necessary assistance according to the actual situation and handle the matter in accordance with relevant regulations. If any accident occurs during the study, please contact the principal investigator in a timely manner.

10. Confidentiality of your personal information

Your personal information, such as your name and contact details, will be kept strictly confidential and will be used only for research records, test arrangements, and necessary research management. Sensory evaluation results will be summarized and analyzed anonymously and will not involve disclosure of personal identity.

11. Must you participate in this study?

Participation in this study is entirely voluntary. You have the right to decide whether to participate, and you also have the right to refuse to continue at any stage after the study begins, without any adverse consequences. Your decision will be fully respected.

12. Can you withdraw from the study midway?

Your participation is entirely voluntary. Even if you have already started the test, you may withdraw at any time without giving a reason, and this will not affect any of your lawful rights and interests.

13. Ethics committee

If you have any questions about this study, you may consult the principal investigator; the telephone number is provided below.

If you have any dissatisfaction or suggestions during the study, please contact the relevant ethics committee or research administration department of the institution.

Telephone: __________

Please keep this document for your records.

Statement of consent

1. I have read this informed consent form, and the responsible person for this project has explained to me in detail the purpose, content, risks, and benefits of this study.
2. I have discussed and asked questions related to this study, and I am satisfied with the answers provided.
3. I have had sufficient time to make my decision.
4. I voluntarily agree to participate in the scientific study described in this informed consent form, and I agree that my research data may be used for the analysis and publication of this study.
5. I agree that representatives of the ethics committee or project funding body may review my research materials when necessary.
6. I will receive a signed and dated copy of this informed consent form.

Finally, I decide to agree to participate in this study.

Participant signature: Date: Year Month Day

Participant contact number:

Participant signature: Date: Year Month Day

Participant contact number:

Participant signature: Date: Year Month Day

Participant contact number:

Participant signature: Date: Year Month Day

Participant contact number:

Participant signature: Date: Year Month Day

Participant contact number:

Participant signature: Date: Year Month Day

Participant contact number:

Participant signature: Date: Year Month Day

Participant contact number:

Participant signature: Date: Year Month Day

Participant contact number:

Participant signature: Date: Year Month Day

Participant contact number:

Participant signature: Date: Year Month Day

Participant contact number:

I confirm that I have explained the details of this study to the participant, including their rights and the possible benefits and risks, and that I have provided them with a signed copy of the informed consent form.

Researcher signature: Date: Year Month Day

Researcher contact information:
