# Supplementary material for: Mining and Validation of Novel Umami Peptides in Non-Alcoholic Beer by Integrating Machine Learning Prediction, Molecular Docking, and Sensory Validation, and Their Multidimensional Sensory Impacts on Beer Body
Source: Foods. 2026 May 11;15(10):1671. doi: 10.3390/foods15101671 (PMC13205247; doi:10.3390/foods15101671)
Supplement: Supplementary file 1 [file foods-15-01671-s001.zip › Supplementary S7.pdf]

**Supplementary S7.** Detailed description of the scoring criteria for different sensory evaluation dimensions of non-alcoholic beer

| Primary dimension | Secondary dimension | Evaluation focus | Specific quantitative rating criteria |
|---|---|---|---|
| Appearance | Color and style match | Assess whether the color matches the declared style, such as pale gold, golden, amber, or dark brown to black, and whether it appears natural and typical | 9 points: Color is highly typical, pure, natural, and highly consistent with the style; 7 points: Basically typical, with slight deviation in shade; 5 points: Acceptable, but style recognizability is average; 3 points: Obvious deviation in color; 1 point: Abnormal color or serious mismatch with the style |
| | Clarity and luster | Assess whether the beer is bright, uniform, and lustrous, and whether any haze falls within the range permitted for the style | 9 points: Clarity or haze level is highly consistent with the style, and the beer is uniform and lustrous; 7 points: Basically, bright and uniform, with slight haziness; 5 points: Acceptable, but brightness is average; 3 points: Noticeably turbid, dull, or non-uniform; 1 point: Severely turbid, with flocculent abnormalities or distorted appearance |
| | Foam color and fineness | Assess whether the foam is white, off-white, or otherwise style-appropriate, and whether the bubbles are fine and uniform | 9 points: Foam color is pure, and bubbles are fine and uniform; 7 points: Foam is relatively fine, with slight coarseness; 5 points: Foam is ordinary, with insufficient uniformity in bubble size; 3 points: Foam is rather coarse or somewhat dark in color; 1 point: Foam is coarse, with obvious gray or yellow discoloration |
| | Foam retention and lacing | Assess foam stability, dissipation rate, and the formation of lacing on the glass wall | 9 points: Foam is stable, dissipates slowly, and forms obvious lacing; 7 points: Foam retention is good, and lacing is fairly clear; 5 points: Acceptable, with moderate dissipation; 3 points: Foam dissipates relatively quickly, with weak lacing; 1 point: Hardly any foam retention and no evident lacing |
| Aroma | Malt aroma | Assess whether malt-related notes such as grain, bread, caramel, and toast are clean, natural, and style-appropriate | 9 points: Malt aroma is clear, natural, layered, and highly consistent with the style; 7 points: Malt aroma is fairly good, but slightly weak or somewhat simple; 5 points: Perceptible, but provides only limited support; 3 points: Malt aroma is weak or insufficiently natural; 1 point: Malt aroma is abnormal, obviously missing, or accompanied by off-notes |
| | Hop aroma | Assess whether floral, herbal, citrus, resinous, or tropical fruit notes are clear, fresh, and not excessively aged | 9 points: Hop aroma is vivid, fresh, typical, and pure; 7 points: Hop aroma is fairly clear, but slightly weak or dull; 5 points: Recognizable, but not prominent; 3 points: Hop aroma is weak, aged, or blurred; 1 point: Hop aroma is seriously distorted or obviously deteriorated |
| | Fermentation character | Assess whether ester, phenolic, acidic, and sulfurous notes are consistent with the yeast profile and style | 9 points: Fermentation character is typical, clean, and highly matched to the style; 7 points: Basically normal, but slightly high or low; 5 points: Acceptable, but character is ordinary; 3 points: Obvious deviation in fermentation character; 1 point: Clear abnormal yeast, sulfur, solvent, or other aromas that should not be present |
| | Aroma complexity and harmony | Assess whether malt, hop, and fermentation aromas show | 9 points: Richly layered, naturally transitioning, and mutually supportive; 7 points: Well balanced, with fairly clear layering; 5 points: Basically harmonious, but with |

| | | | |
|---|---|---|---|
| | | layering and whether they support one another | limited variation; 3 points: One aroma is obtrusive, and layering is poor; 1 point: Serious aromatic imbalance |
| | Cleanliness | Assess whether oxidation, cardboard, stale, sulfurous, moldy-earthy, solvent-like, or other off-aromas are present | 9 points: Very clean, with no perceptible off-aromas; 7 points: Basically clean, with only occasional trace off-notes; 5 points: Slight off-notes are present but still acceptable; 3 points: Off-notes are obvious; 1 point: Clear off-aromas or off-flavors, suggesting a defective sample |
| Taste | Sweetness | Assess whether sweetness provides structural support without becoming cloying | 9 points: Sweetness provides sufficient support, is rounded, and matches the body very well; 7 points: Sweetness is harmonious, but slightly low or high; 5 points: Sweetness is perceptible, but support is average; 3 points: Noticeably thin or cloying; 1 point: Sweetness is severely unbalanced and impairs drinkability |
| | Bitterness | Assess whether bitterness is clean, whether its texture is fine, and whether its intensity matches the style | 9 points: Bitterness is clean and fine-textured, with clear layering and high style consistency; 7 points: Bitterness is fairly good, with slight lingering or slight deviation in intensity; 5 points: Bitterness is present, but quality is average; 3 points: Obvious rough, woody, metallic, or harsh bitterness; 1 point: Bitterness is severely unbalanced and clearly damages the overall profile |
| | Sourness | Assess whether sourness is refreshing, brisk, and natural, and whether it constitutes a defect in non-sour beer styles | 9 points: Sourness is natural, lively, refreshing, and highly consistent with the style; 7 points: Basically natural, but slightly sharp or weak; 5 points: Acceptable; 3 points: Miscellaneous sourness, abrupt sourness, or poor harmony with the style; 1 point: Clear signs of spoilage-like acidity or contamination |
| | Saltiness | Assess whether mineral-like or sea-salt-like salinity is natural, slight, and integrated, noting that most styles should show little to none | 9 points: Saltiness is extremely slight and natural, and can enhance complexity; 7 points: Slightly perceptible and basically integrated; 5 points: Present, but with limited contribution; 3 points: Too strong, rigid, or disruptive to balance; 1 point: Obvious saline-fishy or excessively mineral character, constituting a defect |
| | Umami | Assess whether umami, roundness, or amino-acid-like aftertaste enhances the integrity of the body | 9 points: Umami clearly enhances roundness and aftertaste integrity; 7 points: Provides fairly good savory and smooth support; 5 points: Perceptible, but not prominent; 3 points: Body feels hollow, with insufficient umami support; 1 point: Umami is absent and the structure feels hollow, or abnormal yeast-autolysis-like character appears |
| Drinking experience | Body fullness | Assess whether the body feels thin, full, or heavy, and whether it fits the style | 9 points: Body fullness highly matches the style, being neither thin nor heavy; 7 points: Basically matched, but slightly light or heavy; 5 points: Acceptable; 3 points: Obviously thin or heavy; 1 point: Body is seriously unbalanced |
| | Carbonation bite / carbonation sensation | Assess whether carbon dioxide stimulation is lively and appropriate, and whether it enhances refreshment and aroma release | 9 points: Carbonation bite is lively and appropriate, promoting refreshment and aroma expression; 7 points: Basically appropriate, but slightly strong or weak; 5 points: Acceptable; 3 points: Noticeably too sharp or dull; 1 point: Seriously unbalanced and significantly impairs drinkability |
| | Smoothness / softness | Assess whether the entry is rough, sharp, or palate-scraping, | 9 points: Smooth and soft on entry, without roughness or astringency; 7 points: Basically soft, with slight |

| | | |
|---|---|---|
| | and whether astringency is obvious | angularity; 5 points: Acceptable; 3 points: Obvious palate scraping, roughness, or astringency; 1 point: Strong astringency, burning sensation, or roughness |
| Overall coherence / structure | Assess whether the front, mid, and back palate are coherent, and whether taste, aroma, and carbonation are unified | 9 points: Structure is complete, with natural coherence from front to middle to finish; 7 points: Fairly well coordinated, with slight local abruptness; 5 points: Basically coordinated; 3 points: Loose structure, with disconnection between beginning and finish; 1 point: Severe imbalance |
| Aftertaste cleanliness | Assess whether the finish is clean and free of rough, metallic, or oxidized tailing notes | 9 points: Finish is very clean, with almost no rough tailing notes; 7 points: Fairly clean, with slight tailing; 5 points: Acceptable; 3 points: Rough tailing is rather obvious; 1 point: Clear oxidized, metallic, or stale tailing notes |
| Finish persistence | Assess whether flavor persistence is pleasant, lasting, and not cumbersome | 9 points: Finish is persistent and pleasant, enhancing memorability; 7 points: Fairly good persistence; 5 points: Moderate; 3 points: Poor persistence or unsatisfactory tailing; 1 point: Finish is short and hollow, or lingering in an unpleasant way |
